# Fungi are overlooked dimethylsulfoniopropionate degraders in marine environments

**DOI:** 10.1093/ismejo/wrag269

**Published:** 2026-10-17

**Authors:** Guang-Lei Liu, Min Zhang, Ji-Zheng Song, Yu Wang, Zhao-Lei Zhang, Nan Zhang, Fu-Hui Zheng, Zeng-Tian Gu, Tianjiao Zhu, Ummay Honi, Isabelle Storer, Eva Breyer, Ozlem Pullin, Alice Fredriksson, Stefan Bidula, Federico Baltar, Jonathan D Todd, Yu-Zhong Zhang, Chun-Yang Li

**Affiliations:** MOE Key Laboratory of Evolution and Marine Biodiversity, State Key Laboratory of Marine Food Processing and Safety Control, Frontiers Science Center for Deep Ocean Multispheres and Earth System, Shandong Provincial Key Laboratory of Microbial Resource Exploration and Innovative Utilization & College of Marine Life Sciences, Ocean University of China, Qingdao 266003, China; Marine Biotechnology Research Center, State Key Laboratory of Microbial Technology, Shandong University, Qingdao 266237, China; Laboratory for Marine Biology and Biotechnology, Qingdao Marine Science and Technology Center & Laoshan Laboratory, Qingdao 266373, China; MOE Key Laboratory of Evolution and Marine Biodiversity, State Key Laboratory of Marine Food Processing and Safety Control, Frontiers Science Center for Deep Ocean Multispheres and Earth System, Shandong Provincial Key Laboratory of Microbial Resource Exploration and Innovative Utilization & College of Marine Life Sciences, Ocean University of China, Qingdao 266003, China; MOE Key Laboratory of Evolution and Marine Biodiversity, State Key Laboratory of Marine Food Processing and Safety Control, Frontiers Science Center for Deep Ocean Multispheres and Earth System, Shandong Provincial Key Laboratory of Microbial Resource Exploration and Innovative Utilization & College of Marine Life Sciences, Ocean University of China, Qingdao 266003, China; MOE Key Laboratory of Evolution and Marine Biodiversity, State Key Laboratory of Marine Food Processing and Safety Control, Frontiers Science Center for Deep Ocean Multispheres and Earth System, Shandong Provincial Key Laboratory of Microbial Resource Exploration and Innovative Utilization & College of Marine Life Sciences, Ocean University of China, Qingdao 266003, China; School of Bioengineering, Qilu University of Technology (Shandong Academy of Sciences), Jinan 250353, China; MOE Key Laboratory of Evolution and Marine Biodiversity, State Key Laboratory of Marine Food Processing and Safety Control, Frontiers Science Center for Deep Ocean Multispheres and Earth System, Shandong Provincial Key Laboratory of Microbial Resource Exploration and Innovative Utilization & College of Marine Life Sciences, Ocean University of China, Qingdao 266003, China; Marine Biotechnology Research Center, State Key Laboratory of Microbial Technology, Shandong University, Qingdao 266237, China; Key Laboratory of Marine Drugs Ministry of Education, Ocean University of China, Qingdao 266003, China; School of Biological Sciences, University of East Anglia, Norwich Research Park, Norwich NR4 7TJ, United Kingdom; Centre for Microbial Interactions, Norwich Research Park, Norwich NR4 7TJ. United Kingdom; Centre for Microbial Interactions, Norwich Research Park, Norwich NR4 7TJ. United Kingdom; School of Chemistry, Pharmacy and Pharmacology, University of East Anglia, Norwich Research Park, Norwich NR4 7TJ, United Kingdom; Fungal and Biogeochemical Oceanography Group, College of Oceanography and Ecological Science, Shanghai Ocean University, Nanhui New City, Shanghai 201306, China; School of Biological Sciences, University of East Anglia, Norwich Research Park, Norwich NR4 7TJ, United Kingdom; Centre for Microbial Interactions, Norwich Research Park, Norwich NR4 7TJ. United Kingdom; Quadram Institute, Norwich Research Park, Norwich NR4 7UQ, United Kingdom; School of Biological Sciences, University of East Anglia, Norwich Research Park, Norwich NR4 7TJ, United Kingdom; Centre for Microbial Interactions, Norwich Research Park, Norwich NR4 7TJ. United Kingdom; School of Chemistry, Pharmacy and Pharmacology, University of East Anglia, Norwich Research Park, Norwich NR4 7TJ, United Kingdom; Centre for Microbial Interactions, Norwich Research Park, Norwich NR4 7TJ. United Kingdom; School of Chemistry, Pharmacy and Pharmacology, University of East Anglia, Norwich Research Park, Norwich NR4 7TJ, United Kingdom; Fungal and Biogeochemical Oceanography Group, College of Oceanography and Ecological Science, Shanghai Ocean University, Nanhui New City, Shanghai 201306, China; MOE Key Laboratory of Evolution and Marine Biodiversity, State Key Laboratory of Marine Food Processing and Safety Control, Frontiers Science Center for Deep Ocean Multispheres and Earth System, Shandong Provincial Key Laboratory of Microbial Resource Exploration and Innovative Utilization & College of Marine Life Sciences, Ocean University of China, Qingdao 266003, China; School of Biological Sciences, University of East Anglia, Norwich Research Park, Norwich NR4 7TJ, United Kingdom; Centre for Microbial Interactions, Norwich Research Park, Norwich NR4 7TJ. United Kingdom; Quadram Institute, Norwich Research Park, Norwich NR4 7UQ, United Kingdom; MOE Key Laboratory of Evolution and Marine Biodiversity, State Key Laboratory of Marine Food Processing and Safety Control, Frontiers Science Center for Deep Ocean Multispheres and Earth System, Shandong Provincial Key Laboratory of Microbial Resource Exploration and Innovative Utilization & College of Marine Life Sciences, Ocean University of China, Qingdao 266003, China; Marine Biotechnology Research Center, State Key Laboratory of Microbial Technology, Shandong University, Qingdao 266237, China; Laboratory for Marine Biology and Biotechnology, Qingdao Marine Science and Technology Center & Laoshan Laboratory, Qingdao 266373, China; MOE Key Laboratory of Evolution and Marine Biodiversity, State Key Laboratory of Marine Food Processing and Safety Control, Frontiers Science Center for Deep Ocean Multispheres and Earth System, Shandong Provincial Key Laboratory of Microbial Resource Exploration and Innovative Utilization & College of Marine Life Sciences, Ocean University of China, Qingdao 266003, China

**Keywords:** climate-cooling gases, fungi, marine organosulfur compounds, global sulfur cycling

## Abstract

Dimethylsulfoniopropionate (DMSP) is a key marine organosulfur compound, whose microbial catabolism is critical to global sulfur cycling and is the major source of the climate-active gas dimethyl sulfide (DMS). Research has focused on bacterial and algal DMSP catabolism, potentially overlooking the importance of fungi. Here, we vastly extend the known diversity of fungi that cleave DMSP to DMS, showing that many lacked known DMSP lyases, and Pezizomycotina members were most active. The marine-derived yeast-like fungus *Aureobasidium melanogenum* imported DMSP for osmoprotection, and assimilated it via a previously unreported and efficient cupin-family cytoplasmic DMSP lyase, termed DddF. The DddF co-product, acrylate, was catabolized to acryloyl-CoA in the cytoplasm, with subsequent metabolism occurring in mitochondria. Functional *dddF* genes are widely found across major fungal classes (e.g. Dothideomycetes, Sordariomycetes) common throughout marine ecosystems. Thus, the fungal capacity to catabolize DMSP is far more prevalent than previously believed, highlighting their potential importance in marine sulfur cycling and DMS production.

## Introduction

Each year ~10^9^ tons of the compatible zwitterionic organosulfur molecule dimethylsulfoniopropionate (DMSP) is produced by algae and bacteria in Earth’s surface oceans [1]. Diverse organisms utilize DMSP for important roles in chemotaxis, protection against environmental stresses (e.g. salinity, cold, hydrostatic pressure, and drought), and in nutrient cycling via DMSP catabolism for assimilation and/or respiration [1–6]. Microorganisms can metabolize DMSP via three distinct pathways: DMSP demethylation, oxidation, and cleavage [7, 8]. Only the cleavage pathway releases the climate-cooling gas dimethyl sulfide (DMS), and so far, 11 distinct DMSP lyase enzymes have been identified in diverse microorganisms (Alma1 and DddD/L/Q/W/K/Y/U/P/X/X-like enzymes) [9–11]. In addition, DMSP lyase enzymes also cleave the product of DMSP oxidation, dimethylsulfoxonium propionate (DMSOP), yielding dimethylsulfoxide (DMSO) and a C3 carbon product [8]. DMS, also a key nutrient and signaling molecule in the global oceans, undergoes sea–air exchange and can be oxidized to cloud condensation nuclei that influence the global climate [12–14]. The climate-cooling effect of DMS is estimated to be equivalent in magnitude to the warming effect of anthropogenic CO_2_ emissions [15].

Most research on DMSP cleavage has focused on bacteria (with varied Ddd enzymes), algae, or corals (with the Alma-family DMSP lyase) [11, 16–18]. However, little is known about the diversity and importance of fungal DMSP catabolism, despite their ubiquity in marine environments and their essential role in cycling organic matter and nutrients [19]. Indeed, Earth’s oceans are estimated to harbor ~0.32 Gt of carbon produced by fungi, equivalent to ~20% of the marine prokaryotic biomass (1.6 Gt) [20]. Some filamentous Ascomycete fungi from the genera *Fusarium* and *Aspergillus* cleave DMSP via DddP, an M24 metallopeptidase DMSP lyase [21–23], while a cupin-containing DddW enzyme is also present in *Aspergillus* [24]. However, their DMSP catabolic rates are very low [21, 22, 24], casting doubt on the physiological relevance of DMSP catabolism in these fungal species. Furthermore, the ability of marine yeasts, an abundant and ecologically significant group within the subkingdom Dikarya [19, 20], to utilize DMSP remains unknown, as does their role in global DMSP cycling.

Here, we isolated diverse yeasts and filamentous fungi from mangroves, coastal seawater, and deep-sea sediments and showed that these strains could cleave DMSP, with yeasts displaying much higher activities. The model yeast-like fungus *Aureobasidium melanogenum*, which is a polyextremotolerant species widely distributed in various marine environments [25–27], also showed high DMSP lyase ability. Using functional genomics on *A. melanogenum*, we elucidated the fungal DMSP lyase, termed DddF, together with the downstream catabolic enzymes and mapped their subcellular localization. Furthermore, bioinformatic analysis revealed that *dddF* was widely distributed in diverse fungal taxa common across global oceans, thus highlighting an overlooked role for fungi in marine DMSP cycling.

## Materials and methods

### Isolation, identification, and dimethyl sulfide production of environmental fungi

Fungal strains in this study were isolated from seawater and sediment samples collected from mangrove and coastal areas in China, as well as from deep-sea sediment samples of the Pacific Ocean (Table S1). All samples were suspended in seawater-Yeast Peptone Dextrose (seawater-YPD) medium (20 g/L peptone, 20 g/L glucose, 10 g/L yeast extract prepared in seawater) supplemented with chloramphenicol at a final concentration of 50 mg/L and incubated at 28°C for 48 h [28, 29]. After appropriate dilution of the cell suspensions, the diluted samples were plated on seawater-YPD agar plates containing the same chloramphenicol concentration (50 mg/L), and the plates were incubated at 28°C for 3 days. Distinct colonies were repeatedly subcultured to obtain pure cultures. The internal transcribed spacers (ITS) were amplified from the genomic DNA by polymerase chain reaction (PCR) with primers ITS1 and ITS4 (Table S2) [30], and the resulting amplicons were sequenced for species identification based on sequence alignment using NCBI BLASTn (https://www.ncbi.nlm.nih.gov/). For isolates initially identified as members of the genera *Aspergillus* and *Penicillium* based on ITS sequences, species-level identification was further refined using multilocus phylogenetic analysis (Figs S1 and S2). The ITS, β-tubulin (BenA), and calmodulin (CaM) loci were amplified using the primer pairs ITS1/ITS4, Bt2a/Bt2b, and CMD5/CMD6, respectively (Table S2) [31, 32]. The sequences of these loci were concatenated, and a combined maximum-likelihood phylogeny was constructed as previously described to determine the taxonomic placement of these isolates [33]. To evaluate the growth of purified isolates under saline conditions, we performed additional salt-tolerance assays. Yeast isolates were cultured in YPD medium supplemented with 0 g/L, 35 g/L, and 60 g/L NaCl, respectively, and their growth was monitored by measuring OD_600_. Filamentous fungal isolates were cultured under the same NaCl concentrations at 28°C for 48 h, and their growth was evaluated by harvesting the mycelia via centrifugation, drying to a constant weight, and measuring cell dry weight.

To measure the production rate of DMS, yeast cells were first cultured overnight in NaCl-YPD medium (20 g/L peptone, 20 g/L glucose, 10 g/L yeast extract, and 35 g/L NaCl) at 28°C, harvested by centrifugation, and washed three times using NaCl-Yeast Nitrogen Base (NaCl-YNB) medium (35 g/L NaCl, 5 g/L (NH₄)₂SO₄, 2 mg/L inositol, 40 μg/L CuSO₄, 0.4 mg/L nicotinic acid, 1 g/L KH₂PO₄, 0.5 mg/L H₃BO₃, 0.1 mg/L KI, 0.2 mg/L *p*-aminobenzoic acid, 2 μg/L biotin, 0.5 g/L MgSO₄, 0.2 mg/L FeCl₃, 0.4 mg/L pyridoxine hydrochloride, 2 μg/L calcium pantothenate, 0.4 mg/L MnSO₄, 0.2 mg/L riboflavin, 2 μg/L folic acid, 0.1 g/L CaCl₂, 0.2 mg/L Na₂MoO₄, 0.4 mg/L thiamine hydrochloride, 0.4 mg/L ZnSO₄, Beijing Solarbio Science & Technology Co., Ltd, China). Filamentous fungi cells were also cultured overnight in NaCl-YPD medium at 28°C, and mycelial pellets of filamentous fungi were harvested by filtration through sterile gauze, washed three times with NaCl-YNB medium, and finally resuspended in NaCl-YNB medium for subsequent experiments. The washed cells were added into sealing bottles with 5 mM DMSP at a total volume of 200 μl and incubated at 28°C for 2 h. DMS generation was analyzed with a gas chromatograph (GC-2030, Shimadzu, Japan) equipped with a flame photometric detector. The NaCl-YNB medium with the same concentration of DMSP were set up to monitor the background cleavage of DMSP to DMS, and the same culture of yeast cells without DMSP was set up as the negative control. An eight-point calibration curve of DMSP standards was alkalized with 10 M sodium hydroxide overnight to quantify DMS production. The detection limit was 0.01 nmol. After GC measurement, cells were collected by centrifugation, resuspended in the lysis buffer (50 mM Tris–HCl, 100 mM NaCl, 0.5% glycerol, pH 8.0), and disrupted by ultrasonication. The protein concentration of yeast cells was measured by Pierce BCA Protein Assay Kit (Thermo, USA). DMS production was defined as pmol/min/mg protein.

### Yeast and bacterial strains and plasmids

*Aureobasidium melanogenum* P16, the mutant and complemented strains were cultured in NaCl-YPD medium at 28°C, 180 rpm. The *Escherichia coli* strains BL21 (DE3) and DH5α were cultured in Lysogeny Broth (LB) medium (10 g/L NaCl, 10 g/L tryptone, and 5 g/L yeast extract) at 37°C, 180 rpm. The fungal strains used in this study, which were originally isolated from seawater and sediment samples collected from mangrove and coastal areas in China and from deep-sea sediment samples in the Pacific Ocean, were previously stored in our laboratory. All the strains and plasmids used in this study are listed in Tables S1 and S3.

### Growth of *Aureobasidium melanogenum* P16 grew on dimethylsulfoniopropionate, acrylate, and 3-hydroxypropionate as a carbon source

Cells were first grown in the NaCl-YPD medium at 28°C, 180 rpm for 18 h, and then harvested by centrifugation and washed three times using NaCl-M9 basal medium (6.78 g/L Na_2_HPO_4_, 3 g/L KH_2_PO_4_, 35 g/L NaCl, 1 g/L NH_4_Cl, 0.241 g/L MgSO_4_, 0.011 g/L CaCl_2_). The washed cells were inoculated into the NaCl-M9 basal medium with 5 mM DMSP, acrylate or 3-hydroxypropionate (3-HP) as the sole carbon source, diluted to an initial density of OD_600_ ≈ 0.05 with three replicates, and cultured in the dark at 28°C, 180 rpm. The cells in NaCl-M9 base medium without carbon source were used as the negative control. The growth of the strain was measured by detecting the OD_600_ values of the medium at 24 h intervals using a spectrophotometer V-5100 (METASH, China).

### Genetic manipulations of *Aureobasidium melanogenum* P16

The genetic manipulations of *A. melanogenum* P16 were performed according to our previous study [34]. For gene knockout, the upstream and downstream homologous sequences of the target genes were amplified with specific primers (Table S2). The PCR fragments were inserted to 5′ and 3′ ends of the NAT (nourseothricin resistance gene) cassette of a knockout vector of fl4a-NAT-loxp by Spark HiFi Seamless Cloning Kit (Shandong Sparkjade Biotechnology Co., Ltd, China) to generate knockout plasmids. The linear knockout fragments were amplified from the obtained knockout plasmids using the primers outlined in Table S2 and transferred into competent cells of *A. melanogenum* P16 by electrotransformation. The positive transformants were screened on the YPD agar plates with 100 μg/ml nourseothricin and then were verified by PCR analysis.

For the complementation of target gene mutants, the target gene was amplified by PCR with the primers outlined in Table S2. The PCR fragments were then inserted into the expression vector of pHPTX-loxp with hygromycin resistance gene using HiFi Seamless Cloning Kit (Spark, China). After sequencing validation, the obtained recombinant plasmids were linearized by digestion with *Sma*I and subsequently transformed into the competent cells of *A. melanogenum* P16 by electrotransformation. The positive transformants were screened on the YPD agar plates with 100 μg/ml hygromycin B and then were verified by PCR analysis.

### Quantitative reverse transcription-PCR analysis

*Aureobasidium melanogenum* P16 cells were cultured in NaCl-YPD medium at 28°C for 14 h. Then, the yeast cells were harvested by centrifugation, washed three times using NaCl-YNB medium, and subsequently incubated in NaCl-YNB medium supplemented with 2 mM DMSP for 1 h. Cells grown in NaCl-YNB medium without DMSP were employed as the control group. The total RNA was extracted using an E.Z.N.A. Fungal RNA Kit (Omega, USA), and cDNA was synthesized using a HiScript III RT SuperMix Kit for qPCR (+gDNA wiper) (Vazyme, China). qPCR was performed using ChamQ Universal SYBR qPCR Master Mix (Vazyme, China) on the Rotor-Gene Q real-time fluorescent quantitative PCR analyzer (QIAGEN Hilden, Germany). The translation elongation factor-encoding gene *TEF1* was used as an internal reference gene [35]. The primers used are shown in Table S2.

### Protein expression and purification

The genes were chemically synthesized and then subcloned into the pET-22b and pMAL-c4x vectors (Novagen, USA) by TransGen Biotech (China), and their protein products were overexpressed in *E. coli* BL21 (DE3). The cells were cultured in LB medium with 100 μg/ml ampicillin at 37°C to an OD_600_ of 0.8–1.0, then 0.5 mM isopropyl-β-D-thiogalactopyranoside (IPTG) was added to induce recombinant protein expression for 14 h at 16°C. Cells were harvested by centrifugation (10 min, 7000 rpm, 4°C) and resuspended in lysis buffer (50 mM Tris–HCl, 100 mM NaCl, 0.5% glycerol, pH 8.0), and then disrupted by high-pressure cell cracker. The cell lysates were centrifuged at 10 000 rpm for 1 h at 4°C. The liquid supernatant was added to a Ni^2+^-nitrilotriacetic acid (NTA) column (Cytiva, USA) for affinity chromatography. The recombinant proteins were eluted and subsequently fractionated by gel filtration on a Superdex G200 column (Cytiva, USA). Sodium dodecyl sulfate–polyacrylamide gel electrophoresis (SDS-PAGE) was used to examine the purities of proteins, and protein concentrations were determined by Pierce BCA Protein Assay Kit (Thermo, USA).

### Enzymatic activity assays

To quantify the enzymatic activity of the DddF, 0.01 μM purified DddF enzyme was incubated with 5 mM DMSP, 100 mM Tris–HCl (pH 9.0) at 30°C for 10 min at a total volume of 100 μl, and terminated by adding 10% (v/v) hydrochloric acid. The control groups had the same reaction mixture except that elution buffer was added instead of enzyme. The acrylate production was measured by high-performance liquid chromatography (HPLC) (Ultimate 3000, Dionex and LC-20AT, Shimadzu, Japan) with a Sunfire C18 column (3.5 μm particle size, 4.6 × 250 mm, Waters, USA). The eluent consisted of high-purity water with 2.5% acetonitrile and 0.2% H_3_PO_4_. The samples were eluted at a flow rate of 1 ml/min for 15 min at 30°C, with an injection volume of 10 μl, and monitored via UV absorbance at 210 nm.

To measure the optimal temperature of DddF, the reaction mixture was incubated at 0, 10, 20, 30, 40, and 50°C, and acrylate production was determined as described above. To measure the optimal pH of DddF, the Britton–Robinson Buffer with pH range 5–11 (0.04 M each of phosphoric acid, acetic acid, and boric acid, adjusted to the indicated pH with 0.2 M sodium hydroxide) [35] was added in the reaction mixture. To determine the kinetic parameters of DddF, the reaction mixture containing 1–100 mM DMSP was incubated under optimal temperature and pH and determined by nonlinear analysis based on the initial rates.

### Intracellular concentration measurement

The Δ*dddF* mutant strain was inoculated into NaCl-YPD medium and cultured overnight at 28°C, washed three times with YNB medium, and transferred to NaCl-YNB medium for starvation overnight. Then, the yeast cells were washed and resuspended with 5 mM DMSP and 10 g/L sodium pyruvate in NaCl-YNB medium and cultured for 50 h. Then, 200 μl yeast cells were washed and mixed with 100 μl of 10 M sodium hydroxide for alkaline hydrolysis of intracellular DMSP. Subsequently, 10 μl yeast cells were diluted 100 times and counted with a hemocytometer. To measure the cell volume of *A. melanogenum* P16, the yeast cells carrying the fusion of *dddF* to an *EGFP* gene were examined using a laser scanning confocal microscope (Zeiss LSM900, Germany), with a slice thickness of 1 μm. Subsequently, image analysis was performed using the Imaris software (version 9.0.1) to generate three-dimensional structural models of the cells sampled for the determination of their volumes.

### Liquid chromatography–mass spectrometry analysis

The products of acryloyl-CoA and 3-HP-CoA were measured by liquid chromatography–mass spectrometry (LC–MS) using a Dionex HPLC system with a Bruker impactHD mass spectrometer. Separation was performed on a reversed-phase SunFire C18 column with 1 ml/min flow rate over 24 min. The eluent consisted of high-purity water with 50 mM ammonium acetate (pH 5.5) (solvent A) and 20% acetonitrile with 80% 50 mM ammonium acetate (pH 5.5) (solvent B). A linear gradient was used for separation with 5%–100% solvent B (14 min), 5% solvent B (15 min), and 5% solvent B (24 min) at 30°C. The ionization was processed in electron ionization (EI)-positive mode, and the injection volume was 10 μl.

### Gas chromatography–mass spectrometry analysis

The product 3-HP was measured by gas chromatography–mass spectrometry (GC–MS) with derivatization method [36]. In brief, the reaction was terminated by 10% hydrochloric acid, and the supernatant was mixed with 1 ml chloroform. The sample was vortexed and incubated at 37°C for 45 min, and the organic subaqueous solution was dried in a lyophilizer after centrifugation at 3000 *g* for 30 min. The dried samples were resuspended in 50 μl of methoxyamine hydrochloride (15 mg/ml) in pyridine with incubation at 50°C for 1 h, then silylated with 50 μl of MSTFA (methyl-trimethyl-silyl-trifluoroacetamide) +1% TMCS (Trimethylchlorosilane) (Sigma-Aldrich) at 50°C for 1 h. Separation was performed on a DB-5 MS column (30 m × 0.25 mm × 0.25 μm) with the flow rate of carrier gas (99.9996% helium, Beijing AP Baif Gases Industry Co., Ltd, China) maintained at 1.1 ml/min. The initial oven temperature was held at 60°C for 1 min, then ramped by 10°C/min to 325°C, and kept at 325°C for 10 min. The inlet temperature was set at 250°C. The temperatures of ion source and quadrupole were set at 250°C and 150°C, respectively. The ionization was processed in EI-positive mode. The mass spectra were acquired at a scanning range of 40–600 m/z.

### Subcellular localization

The fusion expression vector pHPT-loxp-MDH3-mCherry was digested with *Sma*I restriction enzyme (MDH3 is a mitochondrial malic enzyme), and the fragment was transformed into *A. melanogenum* P16 to obtain the P16M strain. The target gene and green fluorescent protein (GFP) gene were amplified and inserted into the expression vector pNATX-loxp by Spark HiFi Seamless Cloning Kit (Shandong Sparkjade Biotechnology Co., Ltd, China). The obtained recombinant plasmids were linearized, transferred into the competent cells of *A. melanogenum* P16, and subsequently verified by PCR following the aforementioned protocol. The primers used are shown in Table S2.

As for sample preparation, the fusion expression strains were cultured in YPD agar plates containing 100 μl/ml nourseothricin and hygromycin at 28°C for 48 h. Then, a single colony was inoculated in 5 ml YPD liquid medium, cultured at 180 rpm at 28°C for 20 h, and 5 μl yeast sample were used for observation. The red fluorescence of MDH3-mCherry gene was excited at 587 nm excitation wavelength, and the green fluorescence of DddF-GFP, PrpE-GFP, AcuH-GFP was excited at 488 nm excitation wavelength. Epifluorescence images were taken by a high-resolution confocal laser microscope (Zeiss LSM900, Germany) using a 63 × oil-immersed objective. ZEN software was used to record cell images, and ImageJ software was used for image processing.

### The osmoprotection role of dimethylsulfoniopropionate in *Aureobasidium melanogenum* P16

*Aureobasidium melanogenum* P16 cells were first grown in the NaCl-YPD medium at 28°C, 180 rpm for 18 h, and then harvested by centrifugation and washed three times using NaCl-YNB medium. The washed cells were inoculated into the YNB medium supplemented with 10 g/L sodium pyruvate and NaCl (35 g/L, 60 g/L, and 80 g/L) in the presence and absence of 5 mM DMSP, diluted to an initial density of OD_600_ ≈ 0.05 with three replicates, and cultured in the dark at 28°C, 750 rpm. The growth of the strain was measured at 3 h intervals using a microbial growth curve analyzer MGC-200 (SCIENTZ, China). Quantitative reverse transcription-PCR (RT-qPCR) and DMSP cleavage measurement under salt stress were performed after the fungi cells were cultured in YNB medium supplemented with 10 g/L sodium pyruvate, 5 mM DMSP and NaCl (35 g/L, 60 g/L, and 80 g/L) for 50 h.

### Bioinformatic analysis

To investigate the taxonomic distribution of DddF proteins in fungi, the DddF protein sequence from *A. melanogenum* P16 was used as a query to search the ‘Tara Oceans’ datasets (MATOUv1-metaG) (https://tara-oceans.mio.osupytheas.fr/ocean-gene-atlas/) with an E-value threshold of e^−10^, yielding 48 marine-derived fungal DddF sequences. Subsequently, BLASTP searches were conducted against the NCBI database (data retrieved as of 5 August 2026) across 31 classes within the phylum Ascomycota and 21 classes within the phylum Basidiomycota, restricting the “Organism” field to each class name, with minimum thresholds of 60% query coverage and 40% amino acid sequence identity. After excluding fungal DddF sequences from the species already represented in the ‘Tara Oceans’ datasets, 53 new fungal DddF sequences were obtained. The 101 obtained fungal DddF sequences, together with the DddF sequence from *A. melanogenum* P16, were then used to construct a phylogenetic tree via the maximum-likelihood method with a bootstrap value of 500, implemented using MEGA11 software (https://www. megasoftware.net/). The DddK and DddW sequences from bacteria were employed as an outgroup. The accession numbers of used sequences were shown in Table S4. Sixteen representative DddF homologs were selected for enzymatic activity validation *in vitro* (Fig. S10).

To investigate the geographic distribution of DddF proteins in marine environments, we selected the 16 enzymatically validated sequences, together with one representative sequence per genus from the fungal DddF sequences (Table S4), yielding a final dataset of 38 sequences. Multiple sequence alignment was performed using MUSCLE (v5.3) [37] and trimmed with TrimAl (v1.5.0) [38], and a hidden Markov model (HMM) profile was constructed using HMMER (v3.4, http://hmmer.org/) [39] for homology searches in the ‘Tara Oceans’ dataset (MATOUv1-metaG and MATOUv1-metaT), with an E-value threshold of e^−5^. The relative abundance analyses of *dddP* and *Alma1* were using DddP from *Aspergillus sydowii* (accession number ACV65541.1) [22] and Alma1 from *Emiliania huxleyi* (accession number AKO62592.1) [16] as queries for homology searches with blastp in the ‘Tara Oceans’ dataset (MATOUv1-metaG and MATOUv1-metaT), with an E-value threshold of e^−5^. The relative abundance of *dddF* from the MATOUv1-metaG and MATOUv1-metaT datasets is expressed as percentage of mapped reads. Following the methodology described in previous studies [40], we normalized the results using *TEF1*, a conserved eukaryotic marker [40], to obtain the normalized abundance of DddF in eukaryotic communities in global ocean. Data processing was conducted in Python, and visualization was performed with R [41].

## Results and discussion

### Diverse marine-derived fungi cleave dimethylsulfoniopropionate

To study the diversity of marine fungal DMSP catabolism, 25 marine-derived yeast and 11 filamentous fungal strains were isolated and purified on seawater-YPD medium (lacking DMSP) from mangroves, coastal seawater, and deep-sea sediments. All fungal strains were able to grow under 35 g/L and 60 g/L NaCl (Figs S3 and S4). Despite the omission of DMSP in the isolation media, 63% of the tested isolates (23/36) cleaved this substrate to liberate DMS (Fig. 1 and Table S1). None of the isolates produced methanethiol from DMSP and thus likely did not demethylate DMSP. These data highlighted the natural prevalence and potential importance of fungal DMSP cleavage but not demethylation in natural marine microbial communities. The DMSP catabolic isolates were distributed across the two major fungal phyla, Ascomycota and Basidiomycota, encompassing 11 yeast and 4 filamentous fungal species not previously reported to cleave DMSP (Fig. 1). This representative phylogenetic distribution suggested that DMSP cleavage is prominent in marine-derived fungi. Strains from Pezizomycotina, particularly those of the genus *Aureobasidium*, exhibited stronger DMSP cleavage capability than other tested marine-derived fungi. Furthermore, genomic analysis revealed that these strains, or their relatives with sequenced genomes, lacked homologs with >35% amino acid identity to all characterized DMSP lyase genes, implying that they may possess previously unknown DMSP lyase enzymes.

**Figure 1:**
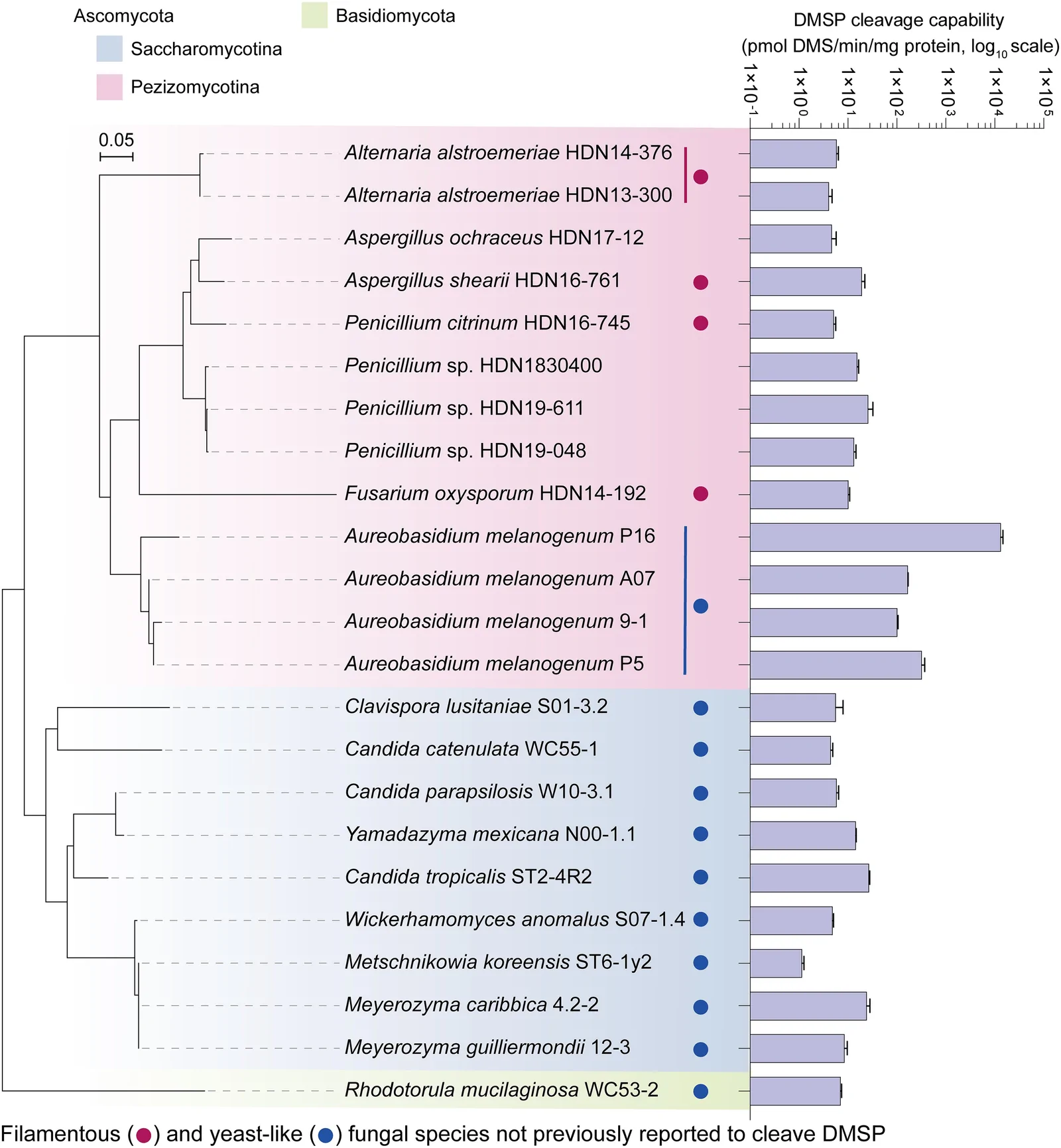
Phylogenetic analysis of marine fungal isolates that cleave DMSP. The DMSP cleavage activity of individual fungal species is shown as horizontal bar plots. Error bars represent standard deviation of triplicate experiments. Fungal strains not previously reported to cleave DMSP are highlighted with circles.

### *Aureobasidium melanogenum* P16 cleaves and assimilates dimethylsulfoniopropionate

To elucidate the DMSP cleavage mechanism in marine-derived fungi, we selected the marine yeast-like fungus *A. melanogenum* P16, previously isolated from Hainan Province mangrove swamps in China [28], as our model organism because it was genome sequenced (accession number GCA_017949455.1) [42], genetically tractable [43], and moreover, we established that it possessed the highest DMSP cleavage ability of all tested strains and grew well on DMSP as sole carbon source (Figs 1, 2A, and S5). These characteristics were not surprising given its host mangrove environments generally possess high DMSP and DMS concentrations [44, 45]. The *A. melanogenum* P16 genome lacked genes with high homology to enzymatically verified DMSP lyases. Only one candidate DMSP lyase was identified (accession number PQ816729), which shared ~30% amino acid sequence identity with bacterial DddK (Table S5). Importantly, this *dddK*-like gene, termed *dddF* hereafter (*ddd* for DMSP-dependent DMS production, and F for fungi), is a member of the cupin superfamily like the DMSP lyases DddK, L, Q, W, Y, and U [17] but is phylogenetically distinct to DddK and all other previously characterized DMSP lyase enzymes (Fig. S6).

**Figure 2:**
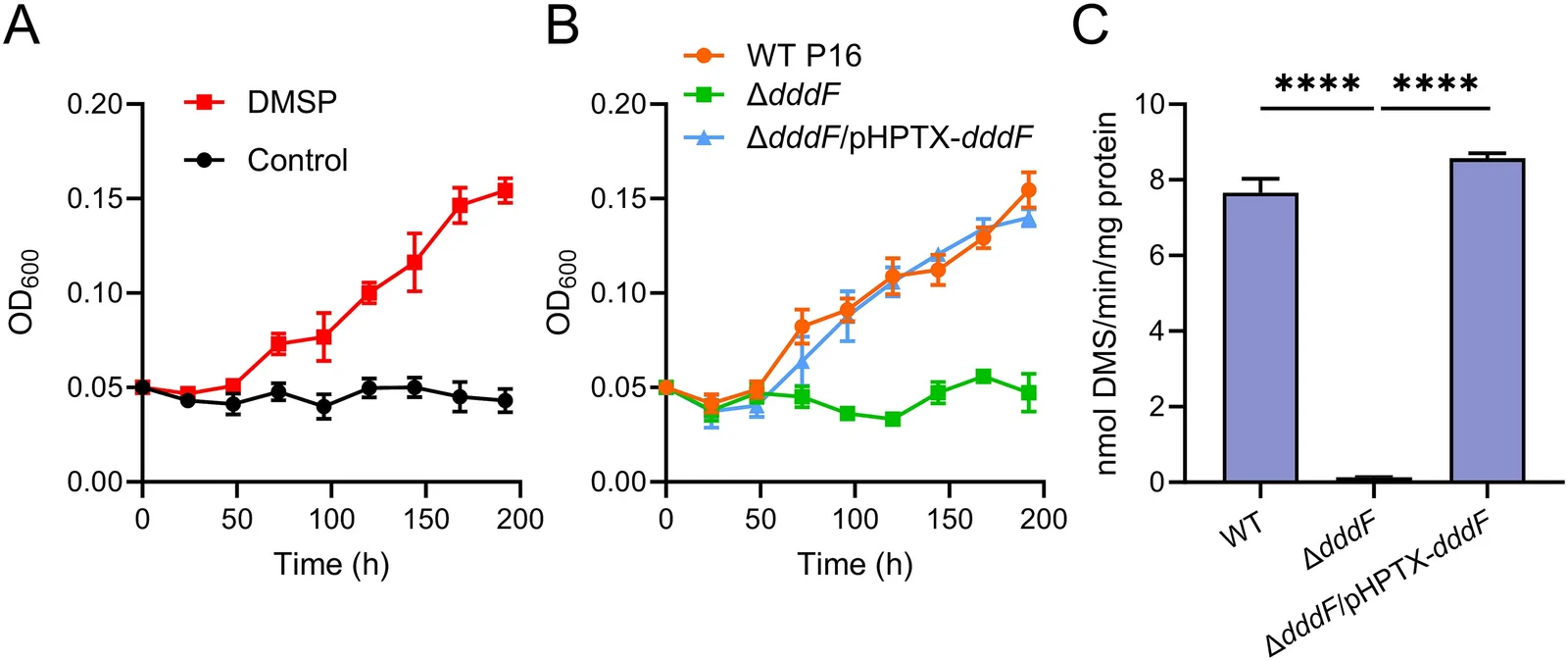
DMSP utilization by *A. melanogenum* P16. (A) Growth curve analysis of strain *A. melanogenum* P16 on DMSP (5 mM) as a sole carbon source. Strain P16 cultured in medium without a carbon source was used as control. (B) Growth curves of wild type *A. melanogenum* P16, Δ*dddF* mutant strain, and the genetically complemented strain Δ*dddF*/pHPTX-*dddF* on DMSP (5 mM) as the sole carbon source. (C) Comparison of DMSP cleavage activity of wild type *A. melanogenum* P16, the Δ*dddF* mutant strain, and the genetically complemented mutant strain Δ*dddF*/pHPTX-*dddF*. Error bars represent standard deviation of triplicate experiments. Statistical significance was analyzed by unpaired two-tailed Student’s *t-*test. ^****^P < 0.0001.

### DddF is the *Aureobasidium melanogenum* P16 dimethylsulfoniopropionate lyase

To verify the *in vivo* function of *dddF*, a Δ*dddF* strain was constructed. This Δ*dddF* strain could no longer utilize DMSP as a carbon source, nor liberate DMS from DMSP (Fig. 2B and C). Growth on DMSP and DMSP-dependent DMS production of the Δ*dddF* strain was restored to almost wild type levels by cloned *dddF* (Fig. 2B and C). These data confirmed that DddF was the DMSP lyase in *A. melanogenum* P16 and essential for the assimilative catabolism of DMSP as a carbon source. RT-qPCR analysis showed that *dddF* transcription was ~3-fold upregulated when *A. melanogenum* P16 was grown with DMSP (Fig. S7), further supporting the role of DddF in DMSP cleavage.

To study *in vitro* DddF DMSP lyase activity, *dddF* was expressed in *E. coli* BL21(DE3), and the recombinant DddF protein was purified. As expected, DddF generated equimolar DMS and acrylate from DMSP (64.8 μmol/s/mg protein). DddF had an optimal temperature of 30°C and pH of 9.0 (Fig. S8A and B). The *K*_M_ of DddF for DMSP was 19.2 mM (Fig. S8C), which was similar in magnitude to other reported bacterial DMSP lyase enzymes [17, 46–48]. This low affinity of DddF for DMSP may facilitate the intracellular accumulation of DMSP, thus allowing its physiological function in e.g. osmoprotection. The *k*_cat_ value of DddF toward DMSP was 45.2 s^−1^, which is ~50 times higher than that of DddK in the ubiquitous marine bacteria *Pelagibacter ubique* HTCC1062 [49–55] (Table S6). These results are reasonable given that the intracellular DMSP concentration of the wild type strain was 576.6 μΜ, while the Δ*dddF* strain accumulated DMSP to 83.1 mM. Not surprisingly, DddF could also cleave DMSOP to DMSO and acrylate like all other reported DMSP lyase enzymes [17] and exhibited a *K*_M_ value of 22.2 mM and *k*_cat_ value of 40.9 s^−1^ for DMSOP (Fig. S8D).

### Acrylate catabolism by *Aureobasidium melanogenum* P16

Like DMSP, *A. melanogenum* P16 was also able to utilize the DMSP cleavage catabolites acrylate and 3-HP as a sole carbon source (Fig. 3A–C). Indeed, the *A. melanogenum* P16 genome contained candidate acrylate catabolic genes encoding PrpE, a propionyl-CoA synthetase that catalyzes the conversion of acrylate to acryloyl-CoA [56], AcuH (an enoyl-CoA hydratase) that converts acryloyl-CoA to 3-hydroxypropionate-CoA (3-HP-CoA) [10], and HACH that catalyzes the formation of 3-HP from 3-HP-CoA (Fig. 3D) (Table S5). To verify the function of these genes, we constructed Δ*prpE*, Δ*acuH*, and Δ*hach* mutants and tested their growth on DMSP, acrylate, and 3-HP. All the mutants could no longer grow on DMSP or acrylate, with growth being complemented by cloned *prpE*, *acuH*, and *hach*. However, the growth of the mutants on 3-HP remained unaffected (Fig. 3A–C). Moreover, purified PrpE, AcuH, and HACH proteins exhibited the expected *in vitro* enzymatic activities (Fig. S9). These data indicated that the *A. melanogenum* P16 PrpE, AcuH, and HACH were *bone fide* propionyl-CoA synthetase, acryloyl-CoA hydratase, and hydroxyacyl-CoA hydrolase, respectively, and were essential for the assimilation of carbon from DMSP and acrylate.

**Figure 3:**
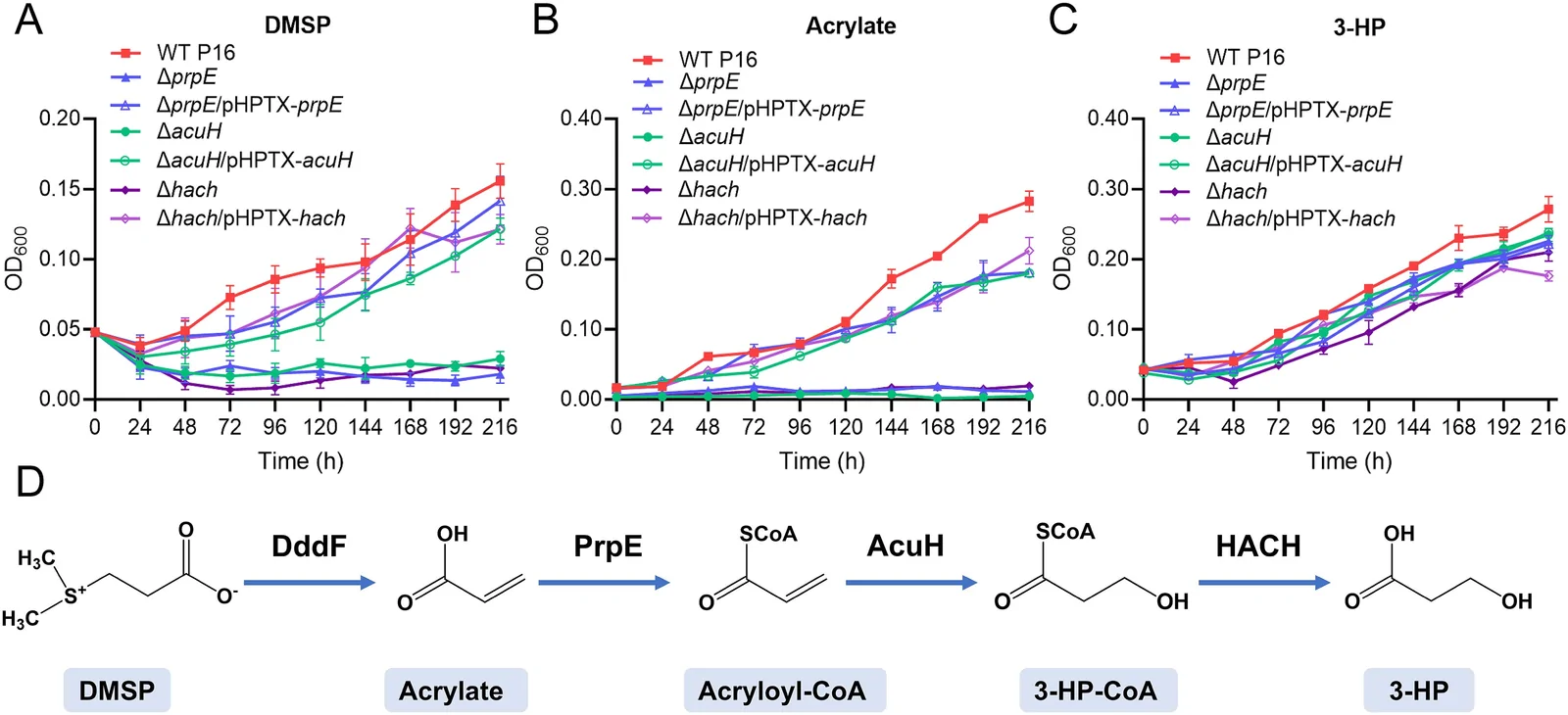
Growth analysis of *A. melanogenum* P16 DMSP catabolic gene mutants. Growth curve analysis of wild type *A. melanogenum* P16, Δ*prpE* mutant, Δ*acuH* mutant, Δ*hach* mutant, and their genetically complemented strains on DMSP (A), acrylate (B), or 3-HP (C) as the sole carbon source. Error bars represent the standard deviation of triplicate experiments. (D) The predicted DMSP catabolism pathway in *A. melanogenum* P16.

The subsequent catabolism of 3-HP has been widely reported in diverse eukaryotes and prokaryotes, as it can be produced from DMSP-independent metabolic pathways [36, 57]. In the nematode *Caenorhabditis elegans*, the HPHD enzyme converts 3-HP to malonate semialdehyde (MalSA) [36]. Genomic analysis showed *A. melanogenum* P16 to contain a HPHD homolog, and also a DddC homolog (Table S5), which may catalyze the conversion of MalSA to acetyl-CoA, and explain how *A. melanogenum* P16 assimilated DMSP.

### Cellular localization of *Aureobasidium melanogenum* P16 dimethylsulfoniopropionate catabolic enzymes

Studies to localize DMSP catabolism in prokaryotes are rare, but such catabolism is generally thought to be cytoplasmic with the exception of some bacteria that contain DddY which can be periplasmic [58]. Such studies are more common in eukaryotes [6, 16, 59], which have organelle differentiation. However, there have been no such localization of DMSP catabolic enzymes in any fungi.

*Aureobasidium melanogenum* P16 DddF and PrpE were predicted to be cytoplasmic enzymes, but AcuH, HACH, HPHD, and DddC were likely mitochondrial (Table S7). To verify these bioinformatic predictions, DddF, PrpE, AcuH, and HACH translational fusions to EGFP were constructed and expressed in *A. melanogenum* P16. The mitochondrial malate dehydrogenase (MDH3) fused to an mCherry tag was used as a control. As indicated in Fig. 4, DddF and PrpE were located in the cytoplasm (Fig. 4A and B), while AcuH and HACH were mitochondrial (Fig. 4C and D). This suggested that DMSP cleavage and acryloyl-CoA synthesis occurred in the cytoplasm, while the subsequent metabolism of acryloyl-CoA likely occurred in the mitochondria (Fig. 5). These localizations are reasonable given that the product of DddC catalysis, acetyl-CoA, feeds into the mitochondrial-located tricarboxylic acid (TCA) cycle; yet, it is currently unclear how acryloyl-CoA would be imported into this organelle.

**Figure 4:**
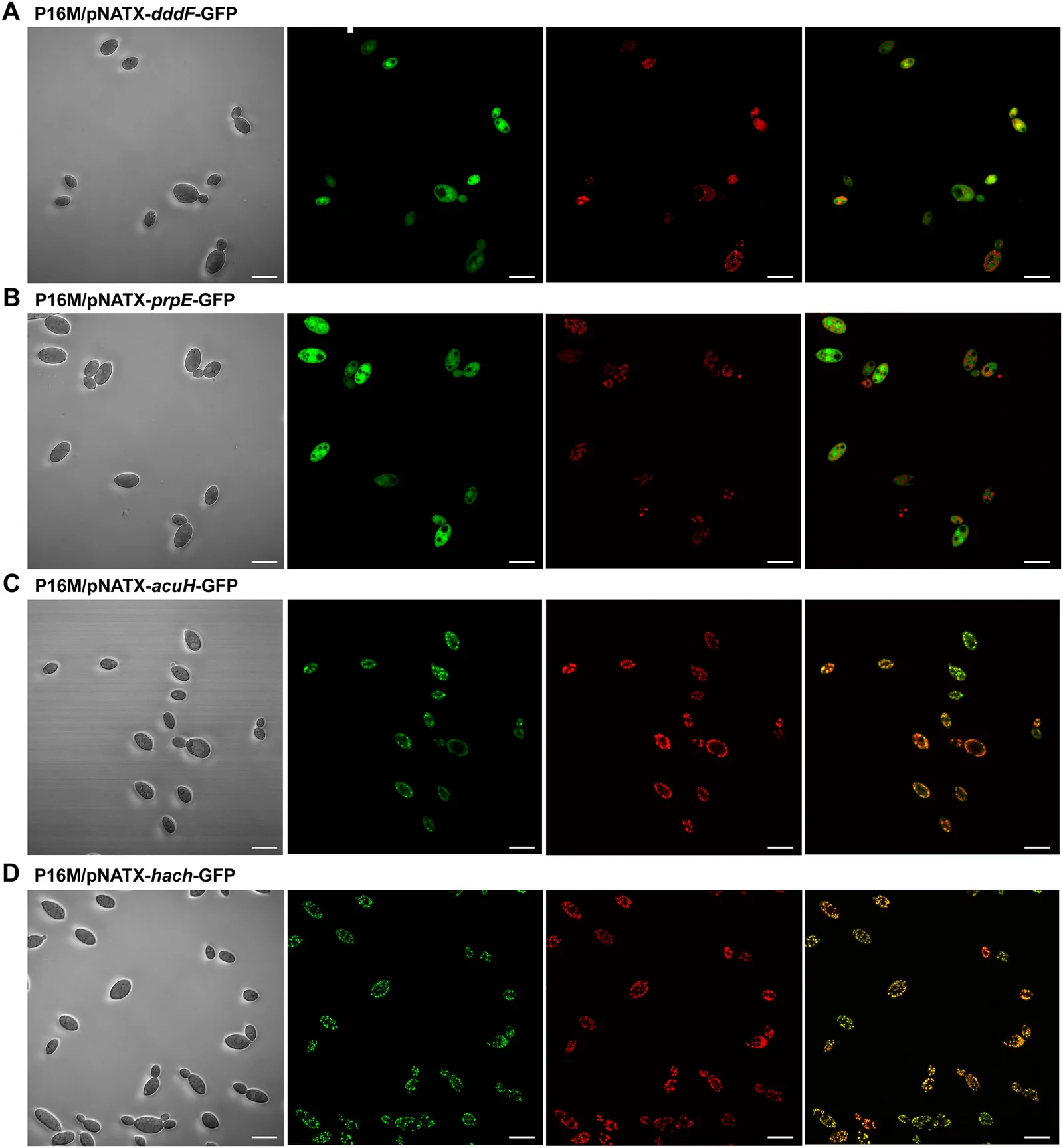
Subcellular localization of DddF (A), PrpE (B), AcuH (C), and HACH (D). The first column shows an *A. melanogenum* P16 cell microscope image. The second column shows green fluorescence protein (GFP) tagged proteins. The third column shows red fluorescence protein (RFP) tagged images of mitochondrial MDH3. The fourth column shows the merging of the GFP and RFP images. Scale bar = 10 μm.

**Figure 5:**
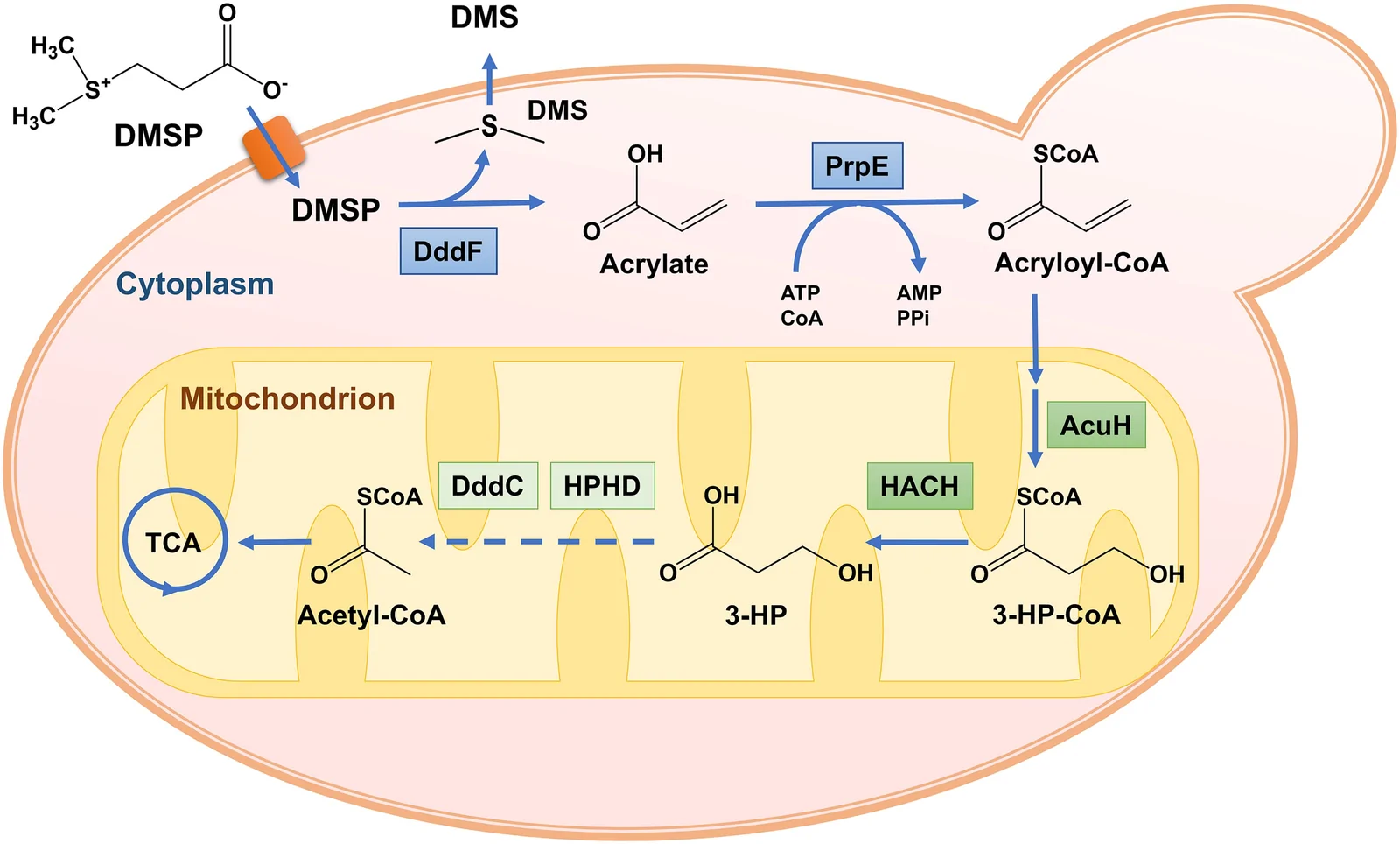
DMSP catabolism in *A. melanogenum* P16. The outer region represents the *A. melanogenum* P16 cytoplasm, while the inner enclosed region represents a mitochondrion. Dotted lines represent unconfirmed steps.

### *Aureobasidium melanogenum* P16 can utilize dimethylsulfoniopropionate as an osmoprotectant

In addition to utilizing DMSP as a sole carbon source, marine bacteria can often accumulate DMSP as an osmoprotectant [60]. For yeasts, it is reported that the main osmolyte accumulating in cells is glycerol, which is produced from the glycolytic intermediate dihydroxyacetone phosphate [61]. To investigate the osmoprotective function of DMSP in *A. melanogenum* P16, we assessed the growth of strain P16 at 35 g/L, 60 g/L, and 80 g/L NaCl in the presence and absence of DMSP. When using pyruvate as a sole carbon source, *A. melanogenum* P16 exhibited a significant growth deficiency at 60 g/L and 80 g/L NaCl, while the supplementation of 5 mM DMSP rescued this growth defect, suggesting the effectiveness of DMSP in relieving NaCl stress in strain P16 (Fig. 6A). In fact, both the transcription of *dddF* and DMSP cleavage capacity of strain P16 were significantly downregulated under gradient salinity (Fig. 6B and C), and the intracellular DMSP concentration of strain P16 was significantly upregulated (Fig. 6D), further supporting that DMSP may be utilized as an osmoprotectant in strain P16.

**Figure 6:**
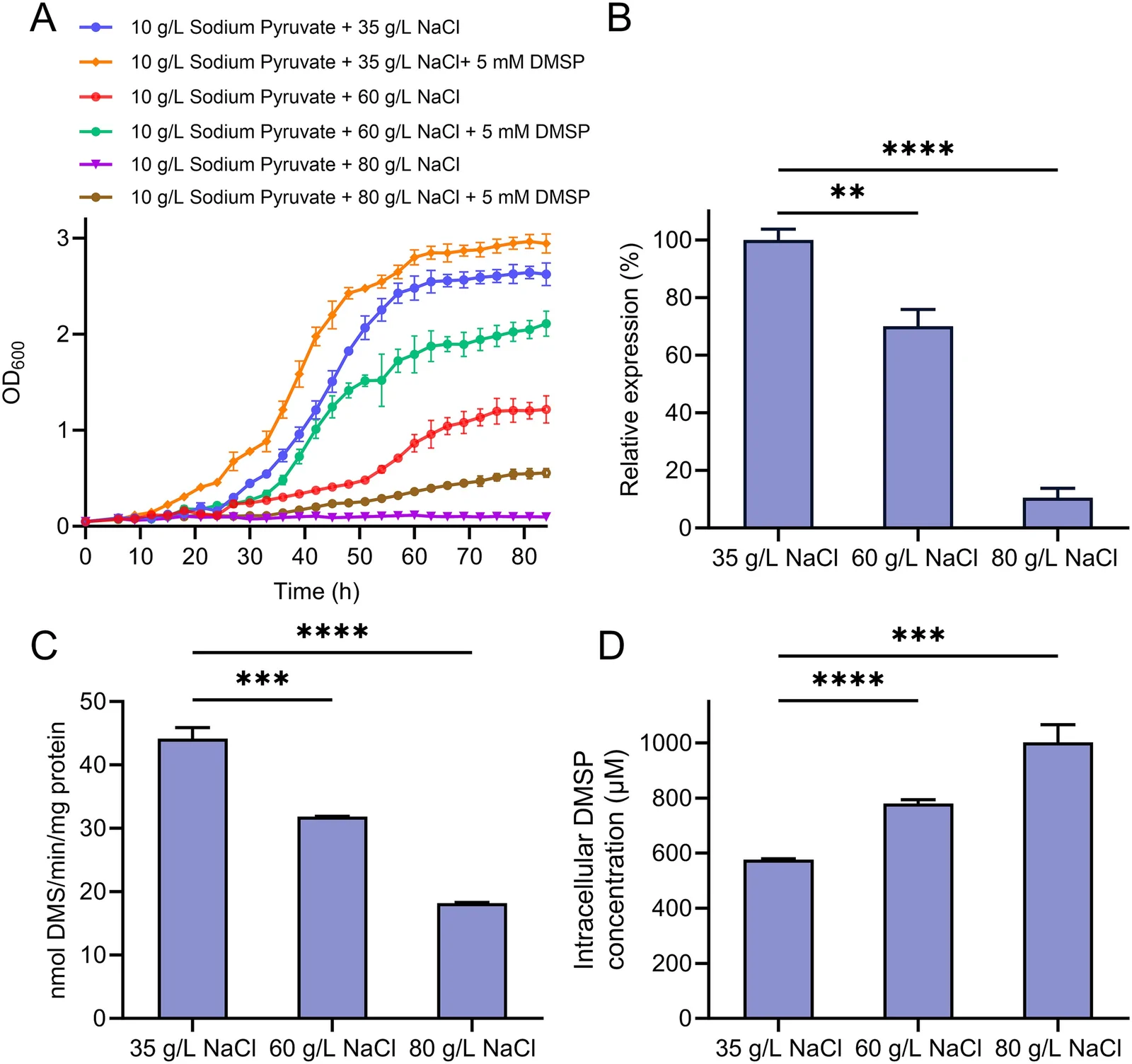
The osmoprotection role of DMSP in *A. melanogenum* P16. (A) Growth curve analysis of strain P16 under salt stress. (B) RT-qPCR analysis of *dddF* transcription under salt stress. (C) DMSP cleavage activity of strain P16 under salt stress. (D) Intracellular DMSP concentration of strain P16 under salt stress. Error bars represent standard deviation of triplicate experiments. Statistical significance was analyzed by unpaired two-tailed Student’s *t-*test. ^*^P < 0.05, ^**^P < 0.01, ^***^P < 0.001, ^****^P <0.0001.

### DddF is widespread in diverse ascomycete fungi

We further studied the taxonomic distribution of *dddF* in fungi. Candidate DddF enzymes, which shared ~40%–90% amino acid identity to *A. melanogenum* P16 DddF, were widely distributed in the Ascomycota subphylum Pezizomycotina, spanning the classes of Dothideomycetes, Eurotiomycetes, Leotiomycetes, Pezizomycetes, and Sordariomycetes, as well as the Saccharomycotina class of Pichiomycetes (Fig. 7A). Moreover, several representative DddF homologs covering the diversity of this protein family were studied and shown to have DMSP lyase activity *in vitro* (Fig. S10). This highlighted that functional DddF homologs were present in diverse and abundant ascomycete fungi. However, no DddF homologs were identified in the Basidiomycota, implying that this fungal phylum may possess other, yet unknown DMSP lyases. In addition, the fungal DddF sequences retrieved from the ‘Tara Oceans’ database were primarily classified within the classes Eurotiomycetes, Dothideomycetes, Sordariomycetes, and Leotiomycetes (Fig. 7A), all of which are reported as the prevalent oceanic fungi of the phylum Ascomycota [19]. Thus, DddF-mediated DMSP cleavage is likely widespread in marine environments.

**Figure 7:**
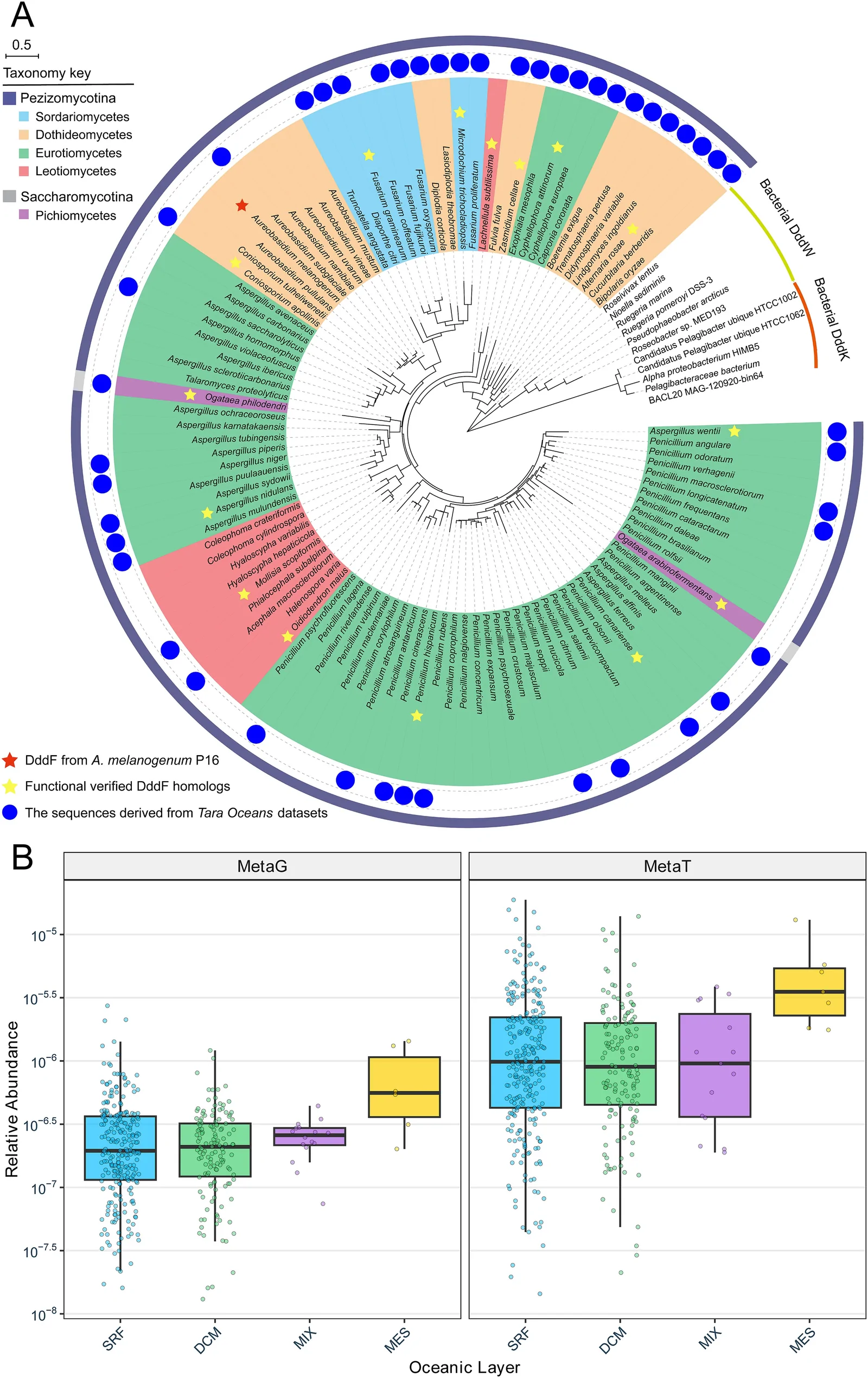
The phylogenetic and abundance analyses of fungal *dddF*. (A) Phylogenetic analysis of fungal DddF with bacterial DddW and DddK sequences. The phylogenetic tree was constructed using maximum-likelihood method with 500 bootstrap replicates in MEGA11. Tree scale = 0.5. (B) The relative abundance of *dddF* in the ‘Tara Oceans’ metagenomes (MATOUv1-metaG) and metatranscriptomes (MATOUv1-metaT). The relative abundance of *dddF* is expressed as percentage of mapped reads. SRF, surface water layer; DCM, deep chlorophyll maximum layer; MIX, epipelagic wind mixed layer; MES, mesopelagic layer; MATOU, Marine Atlas of Tara Ocean Unigenes.

### Distribution and abundance of DddF

To further probe the potential environmental importance of eukaryotic *dddF*, we analyzed its abundance and transcript levels in the global ocean metagenome (metaG) and metatranscriptome (metaT) datasets MATOU v1 from the ‘Tara Oceans’ project [62, 63]. In ‘Tara Oceans’ eukaryotic database, *dddF* and its transcripts were widely distributed within the Atlantic, eastern Pacific, and northwestern Indian Ocean samples (Fig. S11A and B). The *dddF* was found and expressed ubiquitously at similar levels throughout the surface (SRF), deep chlorophyll maximum (DCM), and mixed (MIX) seawater layers. Furthermore, *dddF* and its transcripts were more abundant in the mesopelagic (MES) layer, despite the much lower sample number of the MES layer (Fig. 7B). In addition to *dddF*, the gene and transcript abundances of *dddP* and *Alma1* were also relatively high in marine eukaryotes, indicating that eukaryotes are likely active participants in marine DMSP metabolism (Fig. S12).

To further investigate the proportion of eukaryotes with *dddF*, *dddF* abundance data was normalized using the translation elongation factor-encoding gene *TEF1*, a conserved marker gene for eukaryotes [40]. The normalized abundance of *dddF* varied substantially among different marine vertical layers (Table S8). Both the maximum (3.7%) and minimum (0.02%) normalized abundances of *dddF* were in the SRF layer, indicating the most significant variation in potential fungal DMSP cleavage. The MES layer exhibited the highest mean *dddF* abundance (1.2%), accounting for 0.8%–2.1% of eukaryotes (Table S8), consistent with the *dddF* vertical abundance patterns observed above. Thus, eukaryotic DddF may play important physiological and ecological roles in marine sulfur cycling.

## Conclusion

DMSP is a critically important molecule in global carbon and sulfur cycling. Previous studies on DMSP catabolism have focused on bacteria, algae, and corals, largely overlooking the importance of fungi. Our results here highlight that a greater diversity of marine-derived fungi than previously thought, spanning the classes of Dothideomycetes, Eurotiomycetes, Leotiomycetes, Pezizomycetes, Sordariomycetes, and Pichiomycetes within the phylum Ascomycota—likely assimilate DMSP and utilize it as an osmolyte. These marine-derived fungi contain DddF, one of the most efficient known DMSP lyase enzymes, which enables their assimilation of the DMSP cleavage co-product acrylate. In this way, these marine-derived fungi behave like the commonly studied heterotrophic bacteria with diverse *ddd* genes. Unlike bacteria, however, no fungi have been shown to produce MeSH from DMSP or possess a candidate *dmdA* gene, and the restriction of DMSP demethylation pathway to bacteria remains unexplained. Nevertheless, demonstration that diverse marine-derived fungi can actively import, cleave, and assimilate DMSP fundamentally enhances our understanding of the global sulfur cycle and emphasize that their role should be considered.

## Supplementary Material

supplementary_materials_wrag269

## Data Availability

All data needed to evaluate the conclusions in the paper are present in the paper and/or the Supplementary Materials. All code and the database generated and used in this study have been deposited in Figshare https://doi.org/10.6084/m9.figshare.33016583.
