# Supplementary material for: Fungi are overlooked dimethylsulfoniopropionate degraders in marine environments
Source: ISME J. 2026 Oct 17;20(1):wrag269. doi: 10.1093/ismejo/wrag269 (PMC13738216; doi:10.1093/ismejo/wrag269)
Supplement: Supplementary file 1 [file supplementary_materials_wrag269.docx]

Supplementary Materials for

**Fungi are overlooked dimethylsulfoniopropionate degraders in marine environments**

Guang-Lei Liu, Min Zhang, Ji-Zheng Song, Yu Wang *et al.*

*Corresponding author: Chun-Yang Li, Lcy@ouc.edu.cn.

**This word file includes:**

Figures S1 to S12

Tables S1 to S8

**Fig. S1. The isolated *Aspergillus* strains were identified using a combined phylogenetic analysis based on the concatenated ITS, BenA, and CaM datasets.** Strains isolated in this study are highlighted in bold. The combined phylogenetic tree was constructed using the maximum likelihood method with 500 bootstrap replicates. The GenBank accession numbers following each strain to be identified are listed in the order *ITS*, *BenA* and *CaM*, and the corresponding sequences of the reference strains were obtained from previously described [33]. Based on the combined phylogenetic analysis, strains HDN21-0304 and HDN17-12 were identified as *Aspergillus niveus* and *Aspergillus ochraceus*, respectively.

**Fig. S2. The isolated *Penicillium* strains were identified using a combined phylogenetic analysis based on the** **concatenated ITS, BenA, and CaM datasets.** Strains isolated in this study are highlighted in bold. The combined phylogenetic tree was constructed using the maximum likelihood method with 500 bootstrap replicates. The GenBank accession numbers following each strain to be identified are listed in the order *ITS*, *BenA* and *CaM*, and the corresponding sequences of the reference strains were obtained from previously described [33]. Based on the combined phylogenetic analysis, strains HND14-325, HND16-745, and HND16-761 were identified as *Penicillium brocae*, *Penicillium citrinum*, and *Penicillium shearii*, respectively, whereas strains HDN1830400, HDN19-611, and HDN19-048 were identified only to the genus level, as *Penicillium* sp. HDN1830400, *Penicillium* sp. HDN19-611, and *Penicillium* sp. HDN19-048, due to low bootstrap values that prevented reliable species‑level identification.

**Fig. S3. Growth curves of marine-derived yeasts (A-Y) in YPD medium supplemented with different NaCl concentrations with** ***Saccharomyces cerevisiae* S288C (Z) used as a non-marine yeast control.** All marine-derived yeasts exhibited comparable growth under 0 g/L, 35 g/L and 60 g/L NaCl, whereas growth of the non-marine yeast *S. cerevisiae* S288C was inhibited under 35 g/L and 60 g/L NaCl.

**Fig. S4. Cell dry weight of marine-derived filamentous fungi after 48 h of cultivation in YPD medium supplemented with different NaCl concentrations.** All marine-derived filamentous fungi exhibited comparable growth under 0 g/L, 35 g/L and 60 g/L NaCl.

**Fig. S5. DMSP-dependent DMS production from *A. melanogenum* P16 via GC detection.** The culture medium without yeast and without DMSP were used as the control. The DMS standard was used as a positive control. All chromatograms were offset horizontally (X-axis) and vertically (Y-axis) sequentially to separate curves for better visual distinction, original signal values were not modified.

**Fig. S6. Phylogenetic analysis of DddF and proteins of other experimentally verified cupin-containing DMSP lyases.**

**Fig.** **S7**. **DMSP-induced transcription of *A. melanogenum* P16 *dddF* from RT-qPCR analysis.** The *TEF1* gene was used as an internal reference gene. Error bars represent the standard deviation of triplicate experiments. *****P* < 0.0001.

**Fig. S8**. **Characterization of *A. melanogenum* P16 DddF.** Effects of temperature (A) and pH (B) on DddF activity. (C) A non-linear fit curve for DddF DMSP cleavage. (D) A non-linear fit curve for DddF DMSOP cleavage. The *K*_M_ and *k*_cat_ values are indicated. Error bars represent the standard deviation of triplicate experiments.

**Fig. S9. Detection of *A. melanogenum* P16 PrpE, AcuH and HACH enzymatic activities.** (A) LC-MS analysis of PrpE enzymatic activity. The MW of the main compound is 822.1322, which was equal to that of acryloyl-CoA. (B) LC-MS analysis of AcuH enzymatic activity. The MW of the main compound was 840.1240, which is equal to that of 3-HP-CoA. (C) GS-MS analysis of HACH enzymatic activity. The MW of the main compounds are 73.0467 and 147.0656, which were equal to those of 3-[(Trimethylsilyl)oxy]propionic acid trimethylsilyl ester, derived from 3-HP.

**Fig. S10. Detection of DMSP cleavage activity of DddF homologs.** HPLC detection of the acrylate produced from the cleavage of DMSP by DddF homologs. This result is representative of three triplicate experiments. All chromatograms were offset horizontally (X-axis) and vertically (Y-axis) sequentially to separate curves for better visual distinction, original signal values were not modified.

**Fig. S11.** **Geographic distribution of *dddF* and its transcripts in marine MATOUv1-metaG and** **MATOUv1-metaT**. (A) The geographic distribution of *dddF* (blue symbols). (B) The geographic distribution of *dddF* transcripts (green symbols). The relative abundance of *dddF* is expressed as percentage of mapped reads.

**Fig. S12. The relative abundance of *Alma1* and *dddP* in the *Tara Oceans* metagenomes (MATOUv1-metaG) and metatranscriptomes (MATOUv1-metaT).** The relative abundance of *Alma1* and *dddP* is expressed as percentage of mapped reads. SRF, surface water layer; DCM, deep chlorophyll maximum layer; MIX, epipelagic wind mixed layer; MES, mesopelagic layer; MATOU, Marine Atlas of Tara Ocean Unigenes.

**Table S1. Marine fungi strains used in this study.**

|  | Strains | ITS Accession number | | Source | | Geography | DMSP cleavage capability  (pmol DMS/min/mg protein) | Salinity (PSU) | Temperature (℃) |
| --- | --- | --- | --- | --- | --- | --- | --- | --- | --- |
|  | **Yeast** | | | | | | |  |  |
|  | *Aureobasidium melanogenum* P5 | | PX687880 | Mangrove sediment | | Haikou, China (E110°35'58'', N19°57'28'') | 324.4 ± 48.7 | 23.8 | 30.0 |
|  | *Aureobasidium melanogenum* P16 | | KF531936 | Mangrove seawater | | Haikou, China (E110°35'58'', N19°57'28'') | 13457.4 ± 1407.0 | 23.8 | 30.0 |
|  | *Aureobasidium melanogenum* 9-1 | | KM222352 | Mangrove sediment | | Haikou, China (E110°35'58'', N19°57'28'') | 103.3 ± 2.8 | 23.8 | 30.0 |
|  | *Aureobasidium melanogenum* A07 | | PX687879 | Mangrove seawater | | Haikou, China (E110°35'58'', N19°57'28'') | 168.8 ± 3.0 | 23.8 | 30.0 |
|  | *Candida catenulata* WC55-1 | | PX687876 | Mangrove seawater | | Wenchang, China (E110°49'05'', N19°36'25'') | 4.5 ± 0.4 | 29.9 | 29.8 |
|  | *Yamadazyma mexicana* N00-1.1 | | PX687870 | Mangrove sediment | | Zhanjiang, China (E109°46'47'', N21°31'50'') | 14.5 ± 0.3 | 28.2 | 29.5 |
|  | *Wickerhamomyces anomalus* S07-1.4 | | PX687873 | Coastal seawater | | Shandong, China (E122°25'26'', N36°50'09'') | 4.9 ± 0.2 | 32.3 | 25.5 |
|  | *Rhodotorula dairenensis* ATD1 | | PX687877 | Deepsea sediment | | Pacific Ocean (W134°17'02'', N11°56'01'') | 7.1 ± 0.3 | 34.7 | 3.6 |
|  | *Meyerozyma guilliermondii* 12-3 | | PX687874 | Coastal sediment | | Quanzhou, China (E118°44'35'', N24°50'09'') | 8.5 ± 1.2 | 23.1 | 29.2 |
|  | *Candida tropicalis* ST2-4R2 | PX687871 | | Coastal sediment | | Shantou, China (E116°53'24'', N23°31'12'') | 27.2 ± 0.9 | 34.6 | 29.2 |
|  | *Clavispora lusitaniae* S01-3.2 | PX687872 | | Mangrove seawater | | Zhanjiang, China (E109°46'47'', N21°31'50'') | 5.6 ± 2.3 | 28.2 | 29.5 |
|  | *Meyerozyma caribbica* 4.2-2 | PX872213 | | Mangrove seawater | | Quanzhou, China (E118°44'35'', N24°50'09'') | 24.3 ± 3.9 | 23.1 | 29.2 |
|  | *Candida parapsilosis* W10-3.1 | PX872208 | | Mangrove sediment | | Zhanjiang, China (E109°46'47'', N21°31'50'') | 5.9 ± 0.6 | 28.2 | 29.5 |
|  | *Metschnikowia koreensis* ST6-1y2 | PX872210 | | Mangrove seawater | | Shantou, China (E116°53'24'', N23°31'12'') | 1.2 ± 0.1 | 31.5 | 29.2 |
|  | *Trichosporon asahii* ST3-1Y1 | PX872211 | | Mangrove seawater | | Shantou, China (E116°53'24'', N23°31'12'') | N.D. | 31.5 | 29.2 |
|  | *Saturnispora silvae* ST6-1R2 | PX872212 | | Mangrove seawater | | Shantou, China (E116°53'24'', N23°31'12'') | N.D. | 31.5 | 29.2 |
|  | *Debaryomyces hansenii* N00-2.1 | PX872209 | | Mangrove seawater | | Zhanjiang, China (E109°46'47'', N21°31'50'') | N.D. | 28.2 | 29.5 |
|  | *Candida aaseri* ST3-7R1 | PX872214 | | Mangrove seawater | | Shantou, China (E116°53'24'', N23°31'12'') | N.D. | 31.5 | 29.2 |
|  | *Rhodotorula sphaerocarpa* 7042 | PX872215 | | Coastal seawater | | Yellow Sea (E122°30'35'', N34°43'27'') | N.D. | 30.9 | 26.7 |
|  | Table S1 (Continued) | | | | | | | | |
|  | Strains | ITS Accession number | | Source | | Geography | DMSP cleavage capability  (pmol DMS/min/mg protein) | Salinity (PSU) | Temperature (℃) |
|  | *Rhodotorula glutinis* HK29-3 | PX872216 | | Coastal seawater | | Haikou, China (E110°35'58'', N19°57'28'') | N.D. | 32.5 | 30.0 |
|  | *Rhodotorula mucilaginosa* WC53-2 | PX687878 | | Mangrove seawater | | Wenchang, China (E110°49'05'', N19°36'25'') | N.D. | 29.9 | 29.8 |
|  | *Kodamaea ohmeri* ST3-1y2 | PX687875 | | Coastal sediment | | Shantou, China (E116°53'24'', N23°31'12'') | N.D. | 34.6 | 29.2 |
|  | *Sungouiella intermedia* ST5-6Y1 | PX687869 | | Coastal seawater | | Shantou, China (E116°53'24'', N23°31'12'') | N.D. | 34.6 | 29.2 |
|  | *Trichosporon asahii* S05-1.2 | PX872206 | | Mangrove sediment | | Zhanjiang, China (E109°46'47'', N21°31'50'') | N.D. | 28.2 | 29.5 |
|  | *Candida aaser*i S04-1.1 | PX872207 | | Mangrove sediment | | Zhanjiang, China (E109°46'47'', N21°31'50'') | N.D. | 28.2 | 29.5 |
| **Filamentous fungi** | | | |  | | | |  |  |
| *Alternaria alstroemeriae* HDN13-300 | | PX915494 | | Mangrove root | Haikou, China (E110°35'58'', N19°57'28'') | | 4.1 ± 0.7 | 23.8 | 30.0 |
| *Alternaria alstroemeriae* HDN14-376 | | PX915495 | | Red alga | Qingdao, China (E120°36'47'', N36°04'01'') | | 5.9 ± 0.5 | 33.6 | 25.4 |
| *Aspergillus ochraceus* HDN17-12 | | PZ557045 | | Marine sediment | Pacific Ocean (E143°41'04'', N31°03'40'') | | 4.7 ± 1.1 | 35.2 | 29.6 |
| *Penicillium shearii* HDN16-761 | | PZ557041 | | Marine sediment | Dalian, China (E122°44'46'', N39°00'02'') | | 19.4 ± 3.0 | 32.3 | 24.0 |
| *Fusarium oxysporum* HDN14-192 | | PX915501 | | Marine spongia | South China Sea (E112°02'02'', N16°19'19'') | | 10.3 ± 0.7 | 34.3 | 30.0 |
| *Penicillium* sp. HDN19-611 | | PZ557046 | | Ascidean | Yantai, China (E121°28'18'', N37°36'54'') | | 26.3 ± 6.2 | 32.2 | 24.8 |
| *Penicillium citrinum* HDN16-745 | | PZ557043 | | Marine sediment | Dalian, China (E122°44'46'', N39°00'02'') | | 5.1 ± 0.5 | 32.3 | 24.0 |
| *Penicillium* sp. HDN1830400 | | PZ557040 | | Marine spongia | Weddell Sea, Antarctica (W50°10'17'', S60°14'14'') | | 15.6 ± 1.1 | 34.6 | 3.2 |
| *Penicillium* sp. HDN19-048 | | PZ557047 | | Marine sediment | Mariana Trench (E142°11'58'', N10°20'56'') | | 13.4 ± 1.3 | 34.7 | 2.1 |
| *Penicillium brocae* HDN14-325 | | PZ557042 | | Marine sediment | Indian Ocean (E81°58'41'', N04°41'23'') | | N.D. | 34.8 | 28.7 |
| *Aspergillus niveus* HDN21-0304 | | PZ557044 | | Marine sediment | South China Sea (E119°17'05'', N22°07'02'') | | N.D. | 34.3 | 30.0 |

*: Data are presented as mean ± SD from three independent biological replicates. N.D.: Not Detected.

**Table S2. Primers used in this study.**

| Primer | Sequence | Use |
| --- | --- | --- |
| ITS1 | 5’-TCCGTAGGTGAACCTGCGG-3’ | Amplification of the ITS region |
| ITS4 | 5’-TCCTCCGCTTATTGATATGC-3’ |  |
| Bt2a | 5’-GGTAACCAAATCGGTGCTGCTTTC-3’ | Amplification of the *BenA* gene |
| Bt2b | 5’-ACCCTCAGTGTAGTGACCCTTGGC-3’ |  |
| CMD5 | 5’-CCGAGTACAAGGARGCCTTC-3’ | Amplification of the *CaM* gene |
| CMD6 | 5’-CCGATRGAGGTCATRACGTGG-3’ |  |
| *dddF*-5F | 5’-gattacgccaagcttgcatgcGCTTGGGCAGTGGACGAGC-3’ | Upstream homologous fragment of the *dddF* gene |
| *dddF*-5R | 5’-gctatacgaacggtagtcgacGCGATACGCGATTCAGGAGT-3’ |  |
| *dddF*-3F | 5’-attatacgaacggtaggatccTGGCACACCCTACTCTCAGCTC-3’ | Downstream homologous fragment of the *dddF* gene |
| *dddF*-3R | 5’-acggccagtgaattcgagctcAAGCATCCCACCCGTAGATCT-3’ |  |
| *dddF*-F | 5’-aagatgcatacgcgtgagctcATGTGGTCAGTCTTGCCAAGAAA-3’ | Amplification of the *dddF* gene |
| *dddF*-R | 5’-gctatacgaacggtagtcgacCTAAGCATCCCACCCGTAGATC-3’ |  |
| *dddF*-GF | 5’- acatcaccgtcaaacactagtATGTGGTCAGTCTTGCCAAGAAA-3’ | Amplification of the *dddF* gene |
| *dddF*-GR | 5’- ttgctcaccatAGCATCCCACCCGTAGATCTT-3’ |  |
| EGFP-*dddF*-F | 5’-tgggatgctATGGTGAGCAAGGGCGAGG -3’ | Amplification of EGFP |
| EGFP-*dddF*-R | 5’-gctatacgaacggtagtcgacTTACTTGTACAGCTCGTCCATGCC -3’ |  |
| *prpE*-5F | 5’-TGATTACGCCAAGCTTGCATGCAAGCCAAACAGCGTTCGTA -3’ | Upstream homologous fragment of the *prpE* gene |
| *prpE*-5R | 5’-GTAGTCGACCTGCAGGCATGGTGGTCACAAATGCTCGAGC -3’ |  |
| *prpE*-3F | 5’-AGGATCCCCGGGTACCGAGCTTACACATCTGGCTCGACCGG-3’ | Downstream homologous fragment of the *prpE* gene |
| *prpE*-3R | 5’-GACGGCCAGTGAATTCGAGCTCCGAGGGGAGTGATGACATG-3’ |  |
| *prpE*-F | 5’-acatcaccgtcaaacactagtATGTCTGACGGAAGTGTCAAACC-3’ | Amplification of the *prpE* gene |
| *prpE*-R | 5’-gctatacgaacggtagtcgacTTACTTCTTGCTGGCGTGCA-3’ |  |
| EGFP-*prpE*-F | 5’-acatcaccgtcaaacactagtATGGTGAGCAAGGGCGAGG-3’ | Amplification of EGFP |
| EGFP-*prpE*-R | 5’-acacttccgtcagacatCGAGCCGCCGCCGCCCTT-3’ |  |
| *prpE*-GF | 5’-tcgATGTCTGACGGAAGTGTCAAACC-3’ | Amplification of the *prpE* gene |
| *prpE*-GR | 5’-gctatacgaacggtagtcgacTTACTTCTTGCTGGCGTGCA-3’ |  |
| *acuH*-5F | 5’-GCCAAGCTTGCATGCAAGGAGAAGAAGAGGGAAAG -3’ | Upstream homologous fragment of the *acuH* gene |
| *acuH*-5R | 5’-GACCTGCAGGCATGCCTGTGCAAGATCAAGGACTG -3’ |  |
| *acuH*-3F | 5’-CGAACGGTAGGATCCTAAACGCCCTCTTCTCCCCT-3’ | Downstream homologous fragment of the *acuH* gene |
| *acuH*-3R | 5’-GGTACCCGGGGATCCCCGTGAGGATGATTTCCAT-3’ |  |
| *acuH*-F | 5’-acatcaccgtcaaacactagtATGCAGAGCCTTCGAGTTGC-3’ | Amplification of *acuH* gene |
| *acuH*-R | 5’-gctatacgaacggtagtcgacCTACGAATGCGTCCACTCAGG-3’ |  |
| *acuH*-GF | 5’-acatcaccgtcaaacactagtATGCAGAGCCTTCGAGTTGC-3’ | Amplification of *acuH* gene |
| *acuH*-GR | 5’-ACCATCGAGCCGCCGCCGCCCGAATGCGTCCACTCAGGC-3’ |  |
| EGFP-*acuH*-F | 5’-agcctgagtggacgcattcgGGCGGCGGCGGCTCGATG-3’ | Amplification of the EGFP |
| EGFP-*acuH*-R | 5’-gctatacgaacggtagtcgacTTACTTGTACAGCTCGTCCATGCC-3’ |  |
| Table S2 (Continued) | | |
| Primer | Sequence | Use |
| *hach*-5F | 5’-GATTACGCCAAGCTTGCATGCGTTTCTCATGTAGGCGCCTCC -3’ | Upstream homologous fragment of the *hach* gene |
| *hach*-5R | 5’-GTAGTCGACCTGCAGGCATGCGGATTTCTTAAGCGGGCATGG -3’ |  |
| *hach*-3F | 5’-GGATCCCCGGGTACCGAGCTCGCGAATGTCATCGTTCTCAAGG-3’ | Downstream homologous fragment of the *hach* gene |
| *hach-*3R | 5’-ACGGCCAGTGAATTCGAGCTCGTGGTATTGAACGTCATCGCC-3’ |  |
| *hach*-F | 5’-ACATCACCGTCAAACACTAGTATGCTGCTCCGACCAACTTG-3’ | Amplification of *hach* gene |
| *hach*-R | 5’- gctatacgaacggtagtcgacTCAGTTCCATGAGAAACCTCTCTTG-3’ |  |
| *hach*-GF | 5’-acatcaccgtcaaacactagtATGCTGCTCCGACCAACTTG -3’ | Amplification of *hach* gene |
| *hach*-GR | 5’-ACCATCGAGCCGCCGCCGCCGTTCCATGAGAAACCTCTCTTGTTG -3’ |  |
| EGFP-*hach*-F | 5’- agaggtttctcatggaacGGCGGCGGCGGCTCGATG -3’ | Amplification of the EGFP |
| EGFP-*hach*-R | 5’-gtagtcgacggtaccctgcagTTACTTGTACAGCTCGTCCATGCC-3’ |  |
| DddF-qF | 5’-ACCCAGCCCGAGAGTTTTCC-3’ | Used for RT-qPCR of the *dddF* gene |
| DddF-qR | 5’-TGAGTGTGGCGATGGTGAGC-3’ |  |
| *TEF1*-F | 5’-CAGGTCGGTGCTGGTTAC-3’ | Used for RT-qPCR of the *TEF1* gene |
| *TEF1*-R | 5’-CACATAGGCTTGGAGGGA-3’ |  |

**Table S3.** **Strains and plasmids used in this study.**

| Strains | Description | Source |
| --- | --- | --- |
| *A. melanogenum* P16 | Wild-type isolate | [28] |
| Δ*dddF* | the *dddF* gene deletion mutant | This study |
| Δ*dddF*/pHPTX-*dddF* | Δ*dddF* containing pHPTX-*dddF* plasmid | This study |
| Δ*prpE* | the *prpE* gene deletion mutant | This study |
| Δ*prpE*/pHPTX-*prpE* | Δ*prpE* containing pHPTX-*prpE* plasmid | This study |
| Δ*acuH* | the *acuH* gene deletion mutant | This study |
| Δ*acuH*/pHPTX-*acuH* | Δ*acuH* containing pHPTX-*acuH* plasmid | This study |
| Δ*hach* | the *hach* gene deletion mutant | This study |
| Δ*hach*/pHPTX-*hach* | Δ*hach* containing pHPTX-*hach* plasmid | This study |
| P16M | P16 containing pHPT-loxp-MDH3-mCheery plasmid | This study |
| P16M/pNATX-*dddF*-GFP | P16-mCheery containing pNATX-*dddF*-GFP plasmid | This study |
| P16M/pNATX-*prpE*-GFP | P16-mCheery containing pNATX-*prpE*-GFP plasmid | This study |
| P16M/pNATX-*acuH*-GFP | P16-mCheery containing pNATX-*acuH*-GFP plasmid | This study |
| P16M/pNATX-*hach*-GFP | P16-mCheery containing pNATX-*hach*-GFP plasmid | This study |
| *Escherichia coli* DH5α | Used for gene cloning | Vazyme Biotech company (China) |
| *Escherichia coli* BL21(DE3) | Used for gene expression |  |
| **Plasmids** |  |  |
| fl4a-NAT-loxp* | Used for knockout vector construction | [34] |
| pMD19-T-GFP*** | Used for GFP cloning | This study |
| fl4a-NAT-loxp-Δ*dddF** | Used for the *dddF* gene deletion | This study |
| fl4a-NAT-loxp-Δ*prpE** | Used for the *prpE* gene deletion | This study |
| fl4a-NAT-loxp-Δ*acuH** | Used for the *acuH* gene deletion | This study |
| fl4a-NAT-loxp-Δ*hach** | Used for the *hach* gene deletion | This study |
| pNAT-loxp* | Used for expression vector construction | [34] |
| pHPT-loxp** | Used for expression vector construction |  |
| pHPTX-*dddF*** | Used for the *dddF* gene complementation | This study |
| pHPTX-*prpE*** | Used for the *prpE* gene complementation | This study |
| pHPTX-*acuH*** | Used for the *acuH* gene complementation | This study |
| pHPTX-*hach*** | Used for the *hach* gene complementation | This study |
| pNATX-*dddF-*GFP* | Used for the *dddF*-GFP expression | This study |
| pNATX-*prpE-*GFP* | Used for the *prpE*-GFP expression | This study |
| pNATX-*acuH-*GFP* | Used for the *acuH*-GFP expression | This study |
| pNATX-*hach-*GFP* | Used for the *hach*-GFP expression | This study |
| pMD19-T*** | Used for gene sequencing | TaKaRa (Japan) |
| pHPT-loxp-MDH3-mCheery** | Used for mitochondrial localization | This study |
| pET-22b*** | Used for gene expression | Novagen (Germany) |

* NAT^r^, nourseothricin resistance. ** HPT^r^, hygromycin resistance. ***Amp^r^, ampicillin resistance.

**Table S4. Accession numbers of DddF sequences from different strains.**

| Strains | Accession number |
| --- | --- |
| **Fungi** | |
| **Pezizomycotina** | |
| **Sordariomycetes** | |
| *Microdochium trichocladiopsis** | XP046007895.1 |
| *Diaporthe citri** | XP043017890.1 |
| *Truncatella angustata** | XP045960092.1 |
| *Fusarium oxysporum** | ENH75715.1 |
| *Fusarium graminearum** | XP011321562.1 |
| *Fusarium proliferatum* | XP031074339.1 |
| *Fusarium fujikuroi* | XP023435743.1 |
| *Fusarium coffeatum* | XP031018849.1 |
| **Dothideomycetes** | |
| *Coniosporium tulheliwenetii** | KAJ9637836.1 |
| *Coniosporium apollinis** | XP007785301.1 |
| *Lasiodiplodia theobromae** | XP035372350.1 |
| *Diplodia corticola** | XP020128823.1 |
| *Fulvia fulva** | XP047763064.1 |
| *Zasmidium cellare** | XP033664302.1 |
| *Boeremia exigua** | XP045995626.1 |
| *Trematosphaeria pertusa** | XP033687702.1 |
| *Didymosphaeria variabile** | XP056067592.1 |
| *Lindgomyces ingoldianus** | XP033543601.1 |
| *Alternaria rosae** | XP046026743.1 |
| *Cucurbitaria berberidis** | XP040786209.1 |
| *Bipolaris oryzae** | XP007691738.1 |
| *Aureobasidium melanogenum** | XPA10537.1 |
| *Aureobasidium uvarum* | CAD0106969.1 |
| *Aureobasidium vineae* | CAD0082625.1 |
| *Aureobasidium pullulans* | KAK6005838.1 |
| *Aureobasidium subglaciale* | XP013339736.1 |
| *Aureobasidium namibiae* | XP013423529.1 |
| *Aureobasidium mustum* | CAD0085273.1 |
| **Eurotiomycetes** | |
| *Talaromyces proteolyticus** | XP046068181.1 |
| *Exophiala mesophila** | XP016219757.1 |
| *Capronia coronata** | XP007724362.1 |
| *Cyphellophora europaea** | XP008716672.1 |
| *Cyphellophora attinorum** | XP017999119.1 |
| Table S4 (Continued) | |
| Strains | Accession number |
| *Penicillium canariense** | XP056540412.1 |
| *Penicillium hispanicum** | XP056800987.1 |
| *Penicillium rubens* | XP002558705.1 |
| *Penicillium soppii* | XP057092313.1 |
| *Penicillium citrinum* | KAK5796646.1 |
| *Penicillium crustosum* | CAI7668797.1 |
| *Penicillium longicatenatum* | XP056974500.1 |
| *Penicillium expansum* | KGO64813.1 |
| *Penicillium argentinense* | XP056470061.1 |
| *Penicillium frequentans* | KAJ5525067.1 |
| *Penicillium odoratum* | XP056995668.1 |
| *Penicillium majusculum* | KAJ5694195.1 |
| *Penicillium brevicompactum* | KAJ5367310.1 |
| *Penicillium brasilianum* | CEJ54425.1 |
| *Penicillium riverlandense* | XP057053117.1 |
| *Penicillium corylophilum* | KAN3011965.1 |
| *Penicillium psychrofluorescens* | XP073635648.1 |
| *Penicillium antarcticum* | XP058317764.1 |
| *Penicillium rolfsii* | KAF3394426.1 |
| *Penicillium psychrosexuale* | XP057039104.1 |
| *Penicillium daleae* | XP056764078.1 |
| *Penicillium verhagenii* | KAJ5948599.1 |
| *Penicillium salamii* | CAG8226022.1 |
| *Penicillium coprophilum* | XP056530951.1 |
| *Penicillium maclennaniae* | XP056824884.1 |
| *Penicillium concentricum* | XP056584980.1 |
| *Penicillium nalgiovense* | CAG7951550.1 |
| *Penicillium vulpinum* | OQE10992.1 |
| *Penicillium cataractarum* | XP056551919.1 |
| *Penicillium olsonii* | CAG8136976.1 |
| *Penicillium nucicola* | XP056980226.1 |
| *Penicillium atrosanguineum* | XP056744642.1 |
| *Penicillium manginii* | XP056964499.1 |
| *Penicillium angulare* | XP056772788.1 |
| *Penicillium macrosclerotiorum* | XP056929118.1 |
| *Penicillium cinerascens* | XP058313515.1 |
| *Penicillium lagena* | XP056838939.1 |
| *Aspergillus nidulans** | XP681007.1 |
| Table S4 (Continued) | |
| Strains | Accession number |
| *Aspergillus wentii** | XP040685331.1 |
| *Aspergillus terreus* | KAG2419469.1 |
| *Aspergillus carbonarius* | OOF97384.1 |
| *Aspergillus melleus* | KAK1140083.1 |
| *Aspergillus puulaauensis* | XP041562020.1 |
| *Aspergillus sclerotiicarbonarius* | PYI07225.1 |
| *Aspergillus tubingensis* | OJI83231.1 |
| *Aspergillus saccharolyticus* | XP025430004.1 |
| *Aspergillus avenaceus* | KAE8148745.1 |
| *Aspergillus violaceofuscus* | PYI16263.1 |
| *Aspergillus ibericus* | XP025575036.1 |
| *Aspergillus karnatakaensis* | XP073573944.1 |
| *Aspergillus ochraceoroseus* | XP040756764.1 |
| *Aspergillus homomorphus* | XP025551741.1 |
| *Aspergillus mulundensis* | XP026602088.1 |
| *Aspergillus sydowii* | XP040702058.1 |
| *Aspergillus piperis* | XP025521461.1 |
| *Aspergillus niger* | KAI3003088.1 |
| *Aspergillus affinis* | XP052958813.1 |
| **Leotiomycetes** | |
| *Lachnellula subtilissima** | TVY36379.1 |
| *Mollisia scopiformis** | XP018073234.1 |
| *Acephala macrosclerotiorum** | KAF8848692.1 |
| *Phialocephala subalpina** | CZR55901.1 |
| *Oidiodendron maius** | KIM99310.1 |
| *Halenospora varia** | KAH6667489.1 |
| *Coleophoma crateriformis** | RDW75981.1 |
| *Coleophoma cylindrospora* | RDW82654.1 |
| *Hyaloscypha variabilis** | KAN0092285.1 |
| *Hyaloscypha hepaticicola* | PMD24503.1 |
| **Saccharomycotina** | |
| **Pichiomycetes** | |
| *Ogataea arabinofermentans** | ODV84870.1 |
| *Ogataea philodendri** | XP046062707.1 |
| **Bacteria** | |
| Candidatus Pelagibacter ubique HTCC1002 | EAS84927.1 |
| *Pelagibacteraceae bacterium* BACL20 MAG-120920-bin64 | KRP06000.1 |
| *Alpha proteobacterium* HIMB5 | WP014953073.1 |
| Table S4 (Continued) | |
| Strains | Accession number |
| Candidatus Pelagibacter ubique HTCC1062 | AAZ21215.1 |
| *Roseivivax lentus* | SIS81816.1 |
| *Nioella sediminis* | WP071688972.1 |
| *Ruegeria marina* | SDC47210.1 |
| *Ruegeria pomeroyi* DSS-3 | AAV93771.1 |
| *Pseudophaeobacter arcticus* | WP027241318.1 |
| *Roseobacter* sp. MED193 | EAQ44306.1 |

*Sequences used for hidden Markov model (HMM) construction.

**Table S5.** **Candidate DMSP catabolic proteins in *A. melanogenum* P16.**

| Accession number | Enzymes involved in DMSP and acrylate catabolism | Identity |
| --- | --- | --- |
|  |  |  |
| PQ816729 | DddK (*Pelagibacter ubique* HTCC1062) | 30.9% |
| PQ811709 | PrpE (*Ruegeria lacuscaerulensis* ITI-1157) | 37.2% |
| PQ811707 | AcuH (*Roseovarius nubinhibens* ISM) | 49.9% |
| PO811706 | HACH (*Caenorhabditis elegans*) | 26.6% |
| PV090969 | HPHD (*Caenorhabditis elegans*) | 46.7% |
| PQ816731 | DddC (*Halomonas* sp. HTNK1) | 43.7% |

**Table S6. Kinetic parameters of DMSP** **lyase enzymes.**

| Protein | *K*_M_ for DMSP (mM) | *k*_cat_ for DMSP (s^-1^) | *k*_cat_/*K*_M_ (M^-1^ s^-1^) | Reference |
| --- | --- | --- | --- | --- |
| DddF | 19.2 ± 4.6 | 45.2 ± 3.5 | 2.4 × 10^3^ | This study |
| DddK | 3.7 ± 0.6 | 0.9 ± 0.1 | 2.4 × 10^2^ | [49] |
| DddW | 8.7 ± 0.7 | 18.3 | 2.1 × 10^3^ | [58] |
| DddQ | 21.5 ± 6.8 | 1.0 ± 0.3 | 46.5 | [59] |
| DddX | 0.4 ± 0.03 | 0.7 ± 0.02 | 1.6 × 10^3^ | [48] |
| DddX-like | 1.4 ± 0.05 | 0.42 ± 0.002 | 3.0 × 10^2^ | [11] |
| DddY | 0.4 | ND* | ND | [60] |
|  | 1.4 | ND | ND | [61] |
|  | 5.0 ± 0.6 | 8.3 ± 0.5 × 10^3^ | 1.7 × 10^6^ | [46] |
| Alma1 | 9.0 ± 0.9 | 7 ± 0.3 × 10^2^ | 7.8 ± 0.6 × 10^4^ | [15] |
| DddP | 13.8 ± 5.5 | 0.3 ± 0.1 | 18.7 | [22] |
| DddD | >40** | 12.6 ± 1.5 | 318 | [62] |
| DddU | 8.0 ± 0.8 | 0.7 ± 0.02 | 87.5 | [47] |
| DddL | ND | ND | ND | [63] |

*No data available. **Saturation was not observed at 40 mM.

**Table S7.** **Subcellular localization prediction of *A. melanogenum* P16 proteins involved in DMSP catabolism.**

| Protein | Predicated localization | Probability |
| --- | --- | --- |
| DddF | Cytoplasm | 57.1% |
| PrpE | Cytoplasm | 73.8% |
| AcuH | Mitochondrion | 87.6% |
| HACH | Mitochondrion | 89.8% |
| HPHD | Mitochondrion | 78.3% |
| DddC | Mitochondrion | 87.6% |

**Table S8. Normalized abundance of DddF across global ocean vertical layers in metagenomes.**

| Layer | n | Mean | Median | SD | Min | Max |
| --- | --- | --- | --- | --- | --- | --- |
| SRF | 253 | 0.5% | 0.3% | 0.6% | 0.02% | 3.7% |
| DCM | 134 | 0.6% | 0.3% | 0.5% | 0.02% | 2.3% |
| MIX | 16 | 0.3% | 0.3% | 0.2% | 0.1% | 0.9% |
| MES | 6 | 1.2% | 1.0% | 0.5% | 0.8% | 2.1% |
